# Brucellosis as an Emerging Threat in Developing Economies: Lessons from Nigeria

**DOI:** 10.1371/journal.pntd.0003008

**Published:** 2014-07-24

**Authors:** Marie J. Ducrotoy, Wilson J. Bertu, Reuben A. Ocholi, Amahyel M. Gusi, Ward Bryssinckx, Sue Welburn, Ignacio Moriyón

**Affiliations:** 1 Division of Pathway Medicine and Centre for Infectious Diseases, School of Biomedical Sciences, College of Medicine and Veterinary Medicine, The University of Edinburgh, Chancellor's Building, Edinburgh, United Kingdom; 2 Brucellosis Research Unit, National Veterinary Research Institute, Vom, Plateau State, Nigeria; 3 Avia-GIS, Risschotlei 33, Zoersel, Belgium; 4 Instituto de Salud Tropical y Depto. Microbiología y Parasitología, Universidad de Navarra, Edificio de Investigación, Pamplona, Spain; University of Otago, New Zealand

## Abstract

Nigeria is the most populous country in Africa, has a large proportion of the world's poor livestock keepers, and is a hotspot for neglected zoonoses. A review of the 127 accessible publications on brucellosis in Nigeria reveals only scant and fragmented evidence on its spatial and temporal distribution in different epidemiological contexts. The few bacteriological studies conducted demonstrate the existence of *Brucella abortus* in cattle and sheep, but evidence for *B. melitensis* in small ruminants is dated and unclear. The bulk of the evidence consists of seroprevalence studies, but test standardization and validation are not always adequately described, and misinterpretations exist with regard to sensitivity and/or specificity and ability to identify the infecting *Brucella* species. Despite this, early studies suggest that although brucellosis was endemic in extensive nomadic systems, seroprevalence was low, and brucellosis was not perceived as a real burden; recent studies, however, may reflect a changing trend. Concerning human brucellosis, no studies have identified the *Brucella* species and most reports provide only serological evidence of contact with *Brucella* in the classical risk groups; some suggest brucellosis misdiagnoses as malaria or other febrile conditions. The investigation of a severe outbreak that occurred in the late 1970s describes the emergence of animal and human disease caused by the settling of previously nomadic populations during the Sahelian drought. There appears to be an increasing risk of re-emergence of brucellosis in sub-Saharan Africa, as a result of the co-existence of pastoralist movements and the increase of intensive management resulting from growing urbanization and food demand. Highly contagious zoonoses like brucellosis pose a threat with far-reaching social and political consequences.

## Introduction

Brucellosis is considered one of the most common global zoonoses [1]. Caused by the genus *Brucella* (the most common species being *Brucella abortus*, *B. melitensis*, and *B. suis*), the main clinical signs in animals are abortion and infertility. Brucellosis is highly contagious and is spread through contact with aborted foetuses, vaginal fluids, placentae, placental fluids, and milk, as well as congenitally and venereally. Animals are the only significant source of human brucellosis, and transmission is via direct contact (e.g., veterinarians, abattoir workers, and livestock keepers) and through consumption of unpasteurised dairy products. Human brucellosis is a grave and debilitating disease that may lead to permanent sequelae, requires prolonged and combined antibiotherapy, and is fatal in 1%–5% of untreated cases [2], [3]. Clinical signs are often ignored or incorrectly interpreted, and as a result, human brucellosis is severely underreported [1], [4], [5]. Eradicated in many developed countries after years of effort, brucellosis remains a major neglected zoonosis of low-income nations [1]. Low rates of transmission are typical of brucellosis in extensive systems, and intensification increases the risk of transmission because of higher stocking densities, increased animal contact, and higher birth index [1], [6]–[8]. Increasing co-location of pastoralist nomadism and transhumance with settled and commercial intensive farms may thus create conditions for brucellosis emergence. These circumstances occur in sub-Saharan Africa because of an exceptionally high rural–urban migration caused by the pull of expectation of a better life, and push of unfavourable environmental conditions on agriculture [9], [10].

There is a paucity of science-based evidence on brucellosis in sub-Saharan Africa [1], [4], [11]–[13], and an appraisal of historical and contemporary epidemiology (prevalence estimates, affected host species, potential reservoirs and *Brucella* species) is key to implementing measures for sustainable management of this disease. For a better understanding of these circumstances in the sub-Sahara, we present a review of reports on brucellosis in Nigeria.

Nigeria is the most populous country in Africa (over 170 million in 2012; http://esa.un.org/wpp/ASCII-Data/DISK_NAVIGATION_ASCII.htm) and has an estimated livestock population of 20.49 million cattle, 23.07 million sheep, 28.07 million goats, 6.54 million pigs (http://www.fao.org/ag/againfo/resources/en/glw/GLW_dens.html), 18,200–90,000 camels, and 210,000 horses (http://faostat.fao.org/site/573/default.aspx#ancor) [14]. Nigeria, India, Ethiopia, and Bangladesh account for 44% of poor livestock keepers globally, Nigeria ranking second [8]. Livestock production has always been important in Nigeria, and the rapidly emerging livestock sector now ranks second among the 20 poorest countries [8]. With a large pastoralist population, the livestock industry has been a major focus of government attention since the colonial era (Box 1). Approximately 70% of the population live in rural areas, but there is now considerable rural–urban drift. Increasing demand for animal products has resulted in expansion of animal trade, animal and human movements, and intensification of livestock production systems. The geographic, economic, and social conditions across Nigeria determine the ruminant livestock production systems (Box 2) [15].

**Box 1. Some Events of Significance in the History of Brucellosis in Nigeria**

**Pre-colonial era (before 1900)**

- Livestock production (cattle and small ruminants) dominated by nomadic pastoralism (Fulani) in the savannah region of northern Nigeria. Agricultural land open to grazing post-harvest with mutual benefit of Fulani and farmers (fertilising effect of cow dung).

**British colonial administration**

- **1900–1930.** Tsetse eradication, livestock breeding programmes, and mixed farming approaches. Establishment of Government Veterinary Field and Research Centres (Zaria, 1913; headquarters moved to Vom in 1924; expanded to include vaccine production).
- **1930s.** Government sets up stock farms to improve local breeds (White Fulani, Gudali, and Shuwa). “Mixed Farming Policy” (use of grasslands and pasture by introducing fodder and selected browse plants) to promote agro-pastoralism and range management and livestock productivity.
- **1940s.** Establishment of dairy herds and milk processing plants in Vom and Agege to meet expatriate population demand in Jos and Lagos.

**Independence (1951) to Civil War (1967–1970)**

- **1950s.** Livestock Improvement and Breeding Centres established in Southwest to improve indigenous cattle (humpless dwarf Muturu and Keteku) by crossing with N'dama breed (from Guinea, Sierra Leone, and Congo). N'dama becomes the breed of choice in Southwest (white Fulani remain dominant in the North).
  Western Nigeria Development Corporation established to promote importation of non-autochthonous breeds (South Devon cattle, Friesians, Holsteins, Brown Swiss, Jerseys) to upgrade local stock and increase milk production (most multiplication centres established in the Southwest, with some in the East and North).
  Programmes to encourage settlement of nomadic pastoralists launched (supplementary feeding programme to secure year-round fodder [1962]; grazing reserves [1965 onwards] to protect grazing lands from expanding crop-farms and to resolve clashes over land-use).
- **Early 1960s**. Smallholder steer fattening scheme (Food and Agriculture Organization project) using semi-intensive management systems introduced in the Southwest to ensure supply to local slaughterhouses.

**Post-Civil War to present**

- **Early 1970s.** Nigerian Livestock and Meat Authority established to regulate all aspects of livestock industry and trade. Heavy investments in intensive feedlot fattening for beef.
- **1980s**. Investment in direct livestock production reduces as the government focuses on livestock trade policy and oil industry. Dairy plants set up in Minna, Vom, Kaduna, but inadequate prices cause many to close down.
- **Post-1986.** Government Structural Adjustment Programme Role (GSAPR) in livestock production initiated in 1986 to reform the Nigerian economy, including the livestock sector. The program dwindles, leading to a dominance of the private sector in livestock production. Research institutes (set up in the 1940s) no longer a priority for funding.

**Box 2. Characteristics of Ruminant Livestock Production Systems in Nigeria**

**EXTENSIVE (SUBSISTENCE)**

***North—Pastoral systems (Nomadic or seminomadic)***

 
**Exclusive pastoralist**

1. Livestock only (range, crop residues)
2. Large herds
3. Year-round movements, large range, no permanent homestead

 
**Transhumant**

1. Livestock more than crop (range)
2. Large herds
3. Seasonal migration (quality of grazing and tsetse flies)
4. Permanent homestead

 
**Agro-pastoralists**

1. Livestock more than crop (grazing near environs)
2. Medium-size herds
3. Semi-settled, low-range cattle movements

***South and North—Traditional or village system (sedentary)***

 
**Seasonal tethering**

1. Crop more than livestock (cut-and-carry)
2. Small herds

 
**Fattening**

1. Crop more than livestock (stall feeding)
2. Small herds

 
**Scavenging**

1. Crop more than livestock (scavenging of food scraps in village)
2. Small herds

 
**Compound dairying**

1. Crop more than livestock (stall-feeding or grazing close to homestead)
2. Small herds

**INTENSIVE AND SEMI-INTENSIVE (COMMERCIAL)**

***All areas***

 
**Mixed farming**

1. Crop equals livestock (integrated cropping with livestock rearing)
2. Variable size

***South and North***

 
**Peri-urban and modern husbandry**

1. Livestock only (crop residues, agricultural by-products, grazing)
2. Variable size

The climate varies from semi-arid in the North to tropical in the South. It is estimated that over a third of land that was cultivable 50 years ago is now desert across 11 of Nigeria's northern states and that over 15 million pastoralists are threatened by decreasing access to water and pasture [16]. About half of the semi-arid and sub-humid zones in northern Nigeria are livestock and mixed crop-livestock dominated. Dairy production is concentrated in the North and the beef industry, mostly in the South. Nomadic herdsmen manage about 90% of ruminants and practice seasonal transhumance or year-round nomadism [17], [18]. The Northeast has a hot, dry climate from January to June and rain from June to September. Transhumance is practiced to accommodate variations in available vegetation and agricultural practices and to avoid tsetse flies [19]. In the humid areas of the southern, western, and eastern states, mixed crop-livestock systems dominate, and sheep, goats, and pigs are more important. Pastoralism has been evolving in Nigeria, with farmers often combining cattle production with crop cultivation [20]. Herd sizes have been decreasing as pastoralists are becoming more settled, enabling them to pursue crop farming. Mohammed [21] mentions that a large population of agro-pastoralists settling in the hinterlands of the urban centres in Oyo State were cattle pastoralists displaced from their traditional territories in the North by a variety of agro-ecological and socioeconomic factors. This influx stimulated a new system of livestock production.

The majority (80%) of cattle, mainly Zebu, are concentrated in the savannah zone, with only 10% of the remaining 20% (mostly *Bos taurus*) in the South [15] in a range of management systems (Box 2). Cattle are usually extensively managed, either under nomadic or seminomadic pastoral systems or, to a lesser extent, under traditional village systems, often in contact with small ruminants belonging to the same household. There is more intimate contact between cattle and sheep as they are co-grazed, while goats are left to scavenge free-range. In nomadic systems, small ruminants are sold and exchanged, serving as a “current account,” whereas cattle are traded for status and serve as a “savings account” [22], [23]. Commercial, intensive farms are few and are located on the periphery of major towns in northern and western Nigeria. Cattle reared in extensive systems of the North and the Northeast are transported across Nigeria to the abattoirs of the Southwest to meet the high demand from the economically developed South [24], [25]. According to early reports, 20% of cattle are imported, mostly from Chad and Niger [13].

## Methods

A database search (PubMed, GoogleScholar, Cabdirect, and African Journals Online) was undertaken using broad terms (Brucel* or zoonos* plus Nigeria or Africa) and screened for brucellosis and Nigeria. References in the identified articles were also screened, yielding a total of 164 publications, of which 37 were unobtainable (mostly local journals). Of the remaining 127 publications, 16 were excluded because they were duplicates or were not supported by diagnostic tests. The cattle and small ruminant studies rejected are presented in Tables S1 and S2, respectively.

We used this broad inclusion criterion because (i) only one study (limited to seroprevalence in cattle) met strict scientific criteria and (ii) a critical appraisal of grey literature allowed us to identify presence of the disease, limitations in the use of diagnostic tests, epidemiological aspects, and gaps from which lessons can be drawn. Both the first and corresponding author read all references.

The studies were largely heterogeneous. To summarize their content, we first grouped data by host (cattle, sheep, goats, camels, pigs, horses and donkeys, chickens, dogs, and humans). The data extracted for cattle, small ruminants, and humans are summarised in Tables 1, 2, 3, and 4; Tables S3, S4, S5, S6, S7, S8, S9, S10, S11, S12, S13, S14, S15. Data for other species are discussed in the text (see “Brucellosis in other animals” below). When several hosts were included in the same study, we listed each in the corresponding Table (the common source can be identified in the references cited in the Tables). For cattle and small ruminants, studies were further separated out into farm studies, abattoir or meat market studies, and milk market studies. The farm studies were then further subdivided according to livestock production system (intensive, extensive, or not specified). Where multiple surveys (e.g., abattoir and farm) were reported in a single study, each survey was listed separately. Data were extracted from each reference on:

- population origin,
- sampling method (probability or nonprobability sampling),
- sampling approach (brucellosis investigation, random sampling, multistage sampling, systematic sampling, purposive selection, convenience sampling, etc.),
- diagnostic test used and cut-off (see below),
- bias and/or gaps in sampling method description,
- location of study,
- period of sampling,
- sample size (total number of animals/humans sampled and total number of herds/flocks if information available),
- seroprevalence (individual and herd/flock if available).

**Table 1:** Summary of brucellosis serology studies in cattle in Nigeria.

Row label	Population/*Production system*	Tests, number studies, number individuals and number herds on which INDIVIDUAL PREVALENCE is based					Range of ind prev (%)	Tests, number studies and number. herds on which HERD PREVALENCE is based			Range of herd prev (%)	Refs.
		Tests(number studies)^1^	Number studies^2^	Number individuals^3^	Number studies^4^	Number herds^5^		Tests(number studies)^6^	Number studies^7^	Number herds^8^		
	**Farm**											
**A**	***Intensive***	SAT (6), RBT (4), RPT (2), MRT (1)	13	4341	12	>47	0–47	SAT (5), RBT (4), MRT (1)	10	37	0–100	[25], [29], [32], [47], [53], [54], [97]–[103]
**B**	***Extensive***	RBT (2), MRT (2), MRT/RBT (1)	5	4974	4	>171	2–15	MRT/RBT(1)	1	8	13	[27], [41], [104]–[106]
	***Int/Ext*** 9											
**C**	Intensive	RBT (2), RPT (2), SAT/CFT (1), RBT/ELISA (1)	6	3784	2	>20	3–33	SAT/CFT (1)	1	9	100	[13], [28], [39], [40], [42], [107]
**D**	Extensive	RBT (2), RPT (2), SAT/CFT (1), RBT/ELISA (1)	6	6783	2	>259	0–45 *(41)* 10	SAT/CFT (1)	1	4	0–100	
**E**	***Not specified***	RBT (3), CT/MRT (1)	4	5576	3.5	>199	0–50	RBT (2), CT/MRT (1)	3	134	0–44	[23], [38], [58], [108]
**F**	**Abattoir**	RBT (15), RPT (1), SAT (1)	17	14265	NA	NA	0–22	NA	NA	NA	NA	[13], [40], [58], [108]–[121]
**G**	**Milk Market**	MRT (2)	2	410	NA	NA	7–12	NA	NA	NA	NA	[41], [106]

1 Range of diagnostic tests and respective number of studies for each test on which individual prevalence values in table have been based (see text).

2 Number of studies on which total number of individuals sampled and individual prevalence values have been based.

3 Sum of animal sample size for each study for which individual prevalence data is available.

4 Number of studies, out of total number of studies on which individual prevalence is based, which report number of herds sampled.

5 Minimum estimate of number of herds sampled for each production system category. Not all studies reported number of herds sampled, hence true value must be superior (>) to that in table.

6 Range of diagnostic tests and respective number of studies on which herd prevalence values in table have been based (see text).

7 Number of studies on which total number of herds sampled and herd prevalence values have been based.

8 Sum of number of herds sampled for each study for which herd prevalence data is available.

9 Studies sampling extensive and intensive flocks in parallel.

10 Value of 41% prevalence corresponds prevalence non-adjusted for sensitivity and specificity (apparent prevalence = [true prevalence (0.879+0.998−1)]+1−0.998]; 0.998 = specificity of RBT*ELISA in test series; 0.879 = sensitivity of test series, see Mai et al. 2012).

**Table 2:** Summary of brucellosis serology studies in sheep (S) and goats (G) in Nigeria.

Row label	Population *Production system*	Tests, number studies and number individuals on which INDIVIDUAL PREVALENCE is based						Range of ind prev (%)		Tests, number studies and number flocks on which FLOCK PREVALENCE is based						Range of flock prev (%)		Refs.
		Test (number studies)^1^		Number studies^2^		Number Individuals^3^				Test (number studies)^4^		Number studies 5		Number flocks^6^				
Species		S	G	S	G	S	G	S	G	S	G	S	G	S	G	S	G	
	**Farm**																	
**A**	***Intensive***	RBT (4), RPT (1), SAT (1)	RBT (2), RPT (1)	6	3	594	234	0-76	0-33	RBT (4), SAT (1)	RBT (2)	5	2	5	2	100	100	[45], [47], [53], [101], [122], [123]
**B**	***Extensive***	RBT (1)	RBT (2)	1	2	210	643	5	6-29	NA^8^	NA	0	0	NA	NA	NA	NA	[22], [124]
	***Int/Ext*** 7																	
**C**	Intensive	RBT (2), SAT (1)	RBT (2)	3	2	734	1053	0-21	5-21	NA	NA	0	0	NA	NA	NA	NA	[54]–[56]
**D**	Extensive	RBT (2), SAT (1)	RBT (2)	3	2	570	557	2-13	6-16	NA	NA	0	0	NA	NA	NA	NA	
**E**	***Not specified***	RBT (1)	SAT (2), RBT (1)	1	3	50	985	2	0-5	NA	NA	0	0	NA	NA	NA	NA	[44], [54], [123]
**F**	**Abattoir**	RBT (6), SAT (1)	RBT (8), SAT (2)	7	10	1376	6656	0-15	0-17	NA	NA	NA	NA	NA	NA	NA	NA	[44], [50], [51], [55], [57], [58], [113], [117], [118], [123]

1 Range of diagnostic tests and respective number of studies for each test on which individual prevalence values in table have been based (see text).

2 Number of studies on which total number of individuals sampled and individual prevalence values have been based.

3 Sum of animal sample size for each study for which individual prevalence data is available.

4 Range of diagnostic tests and respective number of studies on which flock prevalence values in table have been based (see text).

5 Number of studies on which total number of flocks sampled and herd prevalence values have been based.

6 Sum of number of herds sampled for each study for which flock prevalence data is available.

7 Studies sampling extensive and intensive flocks in parallel.

8 Not applicable.

**Table 3:** Summary of brucellosis RBT studies in cattle in Nigeria.

Row label	Population/*Production system*	Number studies, number individuals and number herds on which INDIVIDUAL PREVALENCE is based				Range of ind prev (%)	Number studies and number herds on which HERD PREVALENCE is based		Range of herd prev (%)	Refs.
		Number studies^1^	Number individuals^2^	Number studies^3^	Number herds^4^		Number studies^5^	Number herds^6^		
	**Farm**									
**A**	***Intensive***	4	333	4	12	0–33	4	12	0–100	[32], [97], [98], [101]
**B**	***Extensive***	2	3561	2	133	2–16	0	NA^8^	NA	[104], [105]
	***Int/Ext*** 7									
**C**	Intensive	2	152	0	NA	3–8	0	NA	NA	[39], [40], [42]
**D**	Extensive	2	270	0	NA	5–12	0	NA	NA	
**E**	***Not specified***	3	3926	2.5	>174	0–50	2.5	109	0–22	[38], [58], [108]
**F**	**Abattoir**	15	12079	NA	NA	0–22	NA	NA	NA	[40], [58], [108]–[111], [113]–[116], [118]–[121]

1 Number of studies using RBT on which individual prevalence values in table have been based (see text).

2 Sum of animal sample size for each study for which individual prevalence data is available.

3 Number of studies, out of total number of studies, on which individual prevalence is based, which report number of herds sampled.

4 Minimum estimate or true number of herds sampled for each production system category. Not all studies reported number of herds sampled, hence true value must be superior (>) to that in table.

5 Number of studies using RBT on which herd prevalence values in table have been based (see text).

6 Sum of number of herds sampled for each study for which herd prevalence data is available.

7 Studies sampling extensive and intensive flocks in parallel.

8 Not applicable.

**Table 4:** Summary of brucellosis RBT studies in sheep (S) and goats (G) in Nigeria.

Row label	Population *Production system*	Number studies and number individuals on which INDIVIDUAL PREVALENCE is based				Range ind prev (%)		Number studies and number flocks on which FLOCK PREVALENCE is based				Range flock prev (%)		Refs.
		Number studies^1^		Number individuals^2^				Number studies^3^		Number flocks^4^				
Species		S	G	S	G	S	G	S	G	S	G	S	G	
	**Farm**													
**A**	***Intensive***	**4**	**2**	179	124	14–76	21–33	4	2	4	2	100	100	[47], [101], [122], [123]
**B**	***Extensive***	**1**	**2**	210	643	5	6–29	0	0	NA^6^	NA	NA	NA	[22], [123], [124]
	***Int/Ext*** 5													
**C**	Intensive	**2**	**2**	681	1053	0–21	5–21	0	0	NA	NA	NA	NA	[54]–[56]
**D**	Extensive	**2**	**2**	521	557	5–13	6–16	0	0	NA	NA	NA	NA	
**E**	***Not specified***	**1**	**1**	50	28	2	0	0	0	NA	NA	NA	NA	[54]
**F**	**Abattoir**	**6**	**8**	846	3890	0–15	0–17	NA	NA	NA	NA	NA	NA	[50], [51], [55], [57], [58], [113], [118], [123]

1 Number of studies using RBT on which individual prevalence values in table have been based (see text).

2 Sum of animal sample size for each study for which individual prevalence data is available.

3 Number of studies using RBT on which herd prevalence values in table have been based (see text).

4 Sum of number of herds sampled for each study for which herd prevalence data is available.

5 Studies sampling extensive and intensive flocks in parallel.

6 Not applicable.

The intensive farm population (Rows A and C in Tables 1, 2, 3, and 4 and in Tables S3, S5, S10, S12) corresponds to commercial, government or research institutes, and the extensive farm population (Rows B and D in Tables 1, 2, 3, and 4 and in Tables S4, S5, S11, and S12) to Fulani or Indigene (one study only) herds/flocks exclusively. Based on personal field experience in Nigeria, we considered differences in livestock management (for example, nomadic and seminomadic Fulani) across herds of the same category to be of limited significance and merged the values. Studies where the population was not specified were categorised as such (Row E in Tables 1, 2, 3, and 4 and Tables S6 and S13). Some studies conducted surveys in extensively and intensively reared livestock in parallel, and the data for these have been considered separately under Row C and D of Tables 1, 2, 3, and 4 and in Tables S5 and S12. Data from abattoir or meat market studies are summarised in Row F of Tables 1, 2, 3, and 4 (and Tables S7 and S14) and milk market studies in Row G of Table 1 (and Table S8).

**Figure 1:**
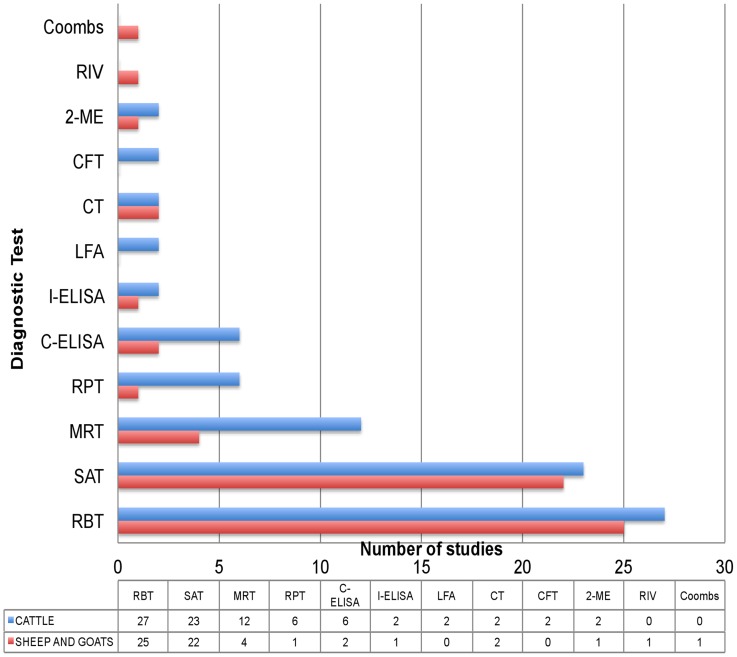
Number of cattle and small ruminant studies which have used the rose Bengal test (RBT), card test (CT), serum agglutination test (SAT), rapid plate test (RPT), 2-mercaptoethanol test (2-ME), rivanol test (RIV), Coombs test, complement fixation test (CFT), milk ring test (MRT), and more recent diagnostic assays such as the competitive ELISA (C-ELISA), indirect ELISA (I-ELISA), and lateral flow assay (LFA) for serological screening. The data table corresponds to total number of studies that have employed each test for each species. The overall number of studies is greater than the total number of papers retrieved because most papers screened sera with more than one serological assay.

Most studies screened sera (blood or milk) with more than one serological assay and therefore report a seroprevalence value based on the results of each individual test. The number of cattle and small ruminant studies which have used classical tests such as the rose Bengal test (RBT), card test (CT), serum agglutination test (SAT), rapid plate test (RPT), 2-mercaptoethanol test (2-ME), rivanol test (RIV), coombs test, complement fixation test (CFT), milk ring test (MRT), and more recent diagnostic assays such as the competitive ELISA (C-ELISA), indirect ELISA (I-ELISA), and lateral flow assay (LFA) are summarised in Figure 1. To summarise and compare data we select one test seroprevalence value per study in this preferential order: RBT (or the equivalent Card Test), CFT, RPT, and SAT (all in blood serum). In studies where only milk was screened with MRT, these values are reported. The rationale for this preferential selection of tests is the superior sensitivity/specificity (in the absence of brucellosis vaccination) of the prioritized tests [26]. Four authors did not report individual test results: Esuruoso [13], who considered samples positive when they were positive for SAT confirmed by CFT for suspicious samples; Alausa [23], who considered samples positive when positive for the card test or MRT or both; Pullan [27], who used MRT screening at herd level and then RBT on individual animals of MRT positive herds; and Mai [28] who confirmed RBT positive or inconclusive samples with C-ELISA. In these cases, we used the positive/negative data provided.

The presentation of average prevalence values calculated from studies using different tests, in different populations, and using different sampling designs is not valid, and so we present only prevalence ranges. We did not average values across analogous livestock production systems using weighting approaches taking into account test performance or sample size because (i) the lack of standardization of tests (origin of antigens, positive and negative controls, cut-off criteria), (ii) the application of brucellosis vaccination in some of the herds tested in earlier studies, and (iii) nonprobability sampling across studies would have led to misleading estimates of average prevalence. These circumstances limit the interpretation of the range of prevalence values presented in Tables 1 and 2. In an attempt to overcome some of these limitations, we consider the RBT values only in Tables 3 and 4, which yield narrower ranges as they are based on fewer studies and a simpler, more robust test, but the overall pattern when comparing intensive and extensive populations is the same (see below).

## Results

### Period of sampling and spatial distribution

**Figure 2:**
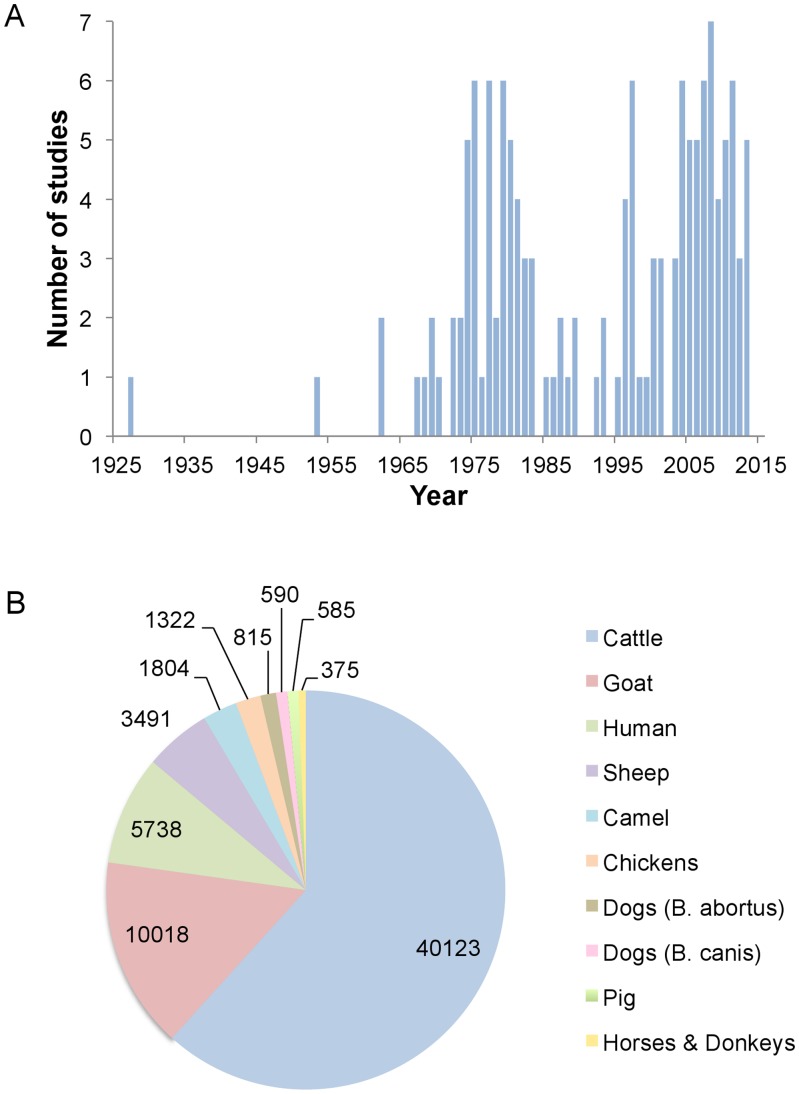
Distribution of studies on brucellosis in Nigeria according to (A) year of publication and (B) host investigated (numbers correspond to cumulative sample size across all studies for each host species).

**Figure 3:**
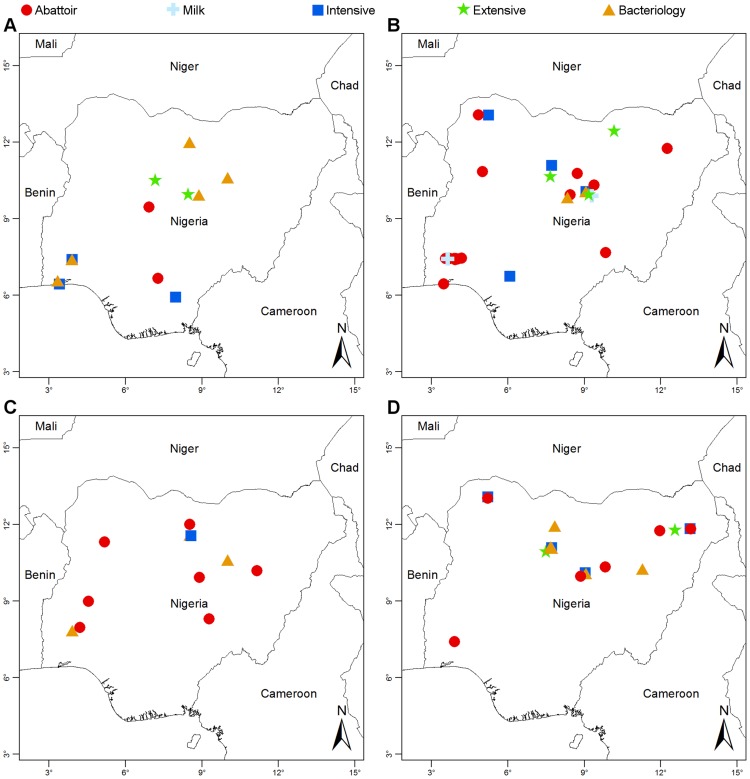
Location of brucellosis studies in Nigeria. (A) cattle; (B) sheep and goats; (C) camels and pigs; and (D) humans.

Historically, two peaks of brucellosis reporting are evident (Figure 2A): the first coincided with establishment of intensive government farms in the 1970s to promote meat production and reduce imports (Box 1); the second with the post-millennium development goals public health agenda, increased interest in neglected zoonotic diseases, and private sector growth. Significantly, the trough coincides with the oil boom of the 1970s (Box 1). Figure 2B shows studies by animal species and Figure 3, the spatial distribution of animal and human studies.

### Cattle brucellosis

To understand brucellosis epidemiology, it is necessary to determine the circulating *Brucella* species and biovars and, as antibodies are not species specific, bacterial isolation is essential. Since brucellosis was first reported in Nigeria in 1927 [29], only five studies have provided bacteriological data for cattle (Figure 3). In the West, studies in range cattle and in a University herd described the isolation of *Brucella* strains, probably *B. abortus* [30]. This species was properly identified in studies in government and private farms and in settled Fulani herds in the Centre and North [31]–[33]. In total, 58 isolates were classified as *B. abortus* biovar 1 (54 strains), biovar 2 (1 strain), biovar 3 (2 strains), and biovar 4 (1 strain) (see Table S9). However, re-examination of 20 of the biovar 1 isolates shows characteristics of biovar 3, the dominant biovar in countries proximal to Nigeria [34]. Moreover, VNTR genotyping [35] clusters these 20 strains with biovar 3a rather than 3b, the latter being typically reported in Europe (Ducrotoy, Bertu, Moriyón, and Ocholi, unpublished results). *B. melitensis* has not been reported in cattle, although there is close contact with small ruminants.

The bulk of the evidence is derived from serological studies (Figure 1), but limitations in the application of serological tests make data difficult to interpret. Early studies used RPT or SAT, two tests lacking sensitivity and specificity [26], [36], [37]. The RBT (or the equivalent Card Test) was applied shortly after its development and has been widely used (Tables 1 and 3; Figure 1). Despite the excellent specificity and sensitivity of RBT [26], [36], [37], the literature reviewed reflects the misconception that RBT is a test of low specificity which, in the absence of brucellosis vaccination or the false positive serological reaction phenomenon caused by crossreacting bacteria, needs to be confirmed. However, meta-analysis performed using strict criteria [26] shows that RBT specificity is in fact better than that of iELISA and cELISA, two tests used in some works to “confirm” the RBT results. Indeed, the OIE Manual (http://www.oie.int/en/international-standard-setting/terrestrial-manual/access-online/; Chapter 2.4.3. Bovine Brucellosis) clearly states that these other tests can also sometimes give a positive result because of S19 vaccination or of false-positive serological reactions.

While RBT is a good choice, inadequate standardization results in considerable sensitivity (but not specificity) variation [37]. RBT standardization and origin was inadequately described in 15 out of 46 papers and six investigations used locally prepared antigens. Competitive or indirect ELISA kits were used according to manufacturer instructions but were never validated under local conditions (cut-offs established in brucellosis-free and good hygienic conditions cannot be extrapolated to endemic areas [38]).

Across Nigeria, 14,000, 11,000, and 8,000 cattle have been sampled in different studies from abattoirs (animals from both extensive and intensive systems), extensive, and intensive herds, respectively, but the data (Tables 1 and 3; Tables S3, S4, S5, S6, S7, S8, S9; Figures 2A and 3A) illustrate the limitations in time and space of the studies. A total of 1,800 cattle correspond to the North, half this number (1,000) to the West and only small numbers to the East and South. Abattoir studies cannot provide spatial information due to country-wide animal movements (see above). Only five out of the 46 prevalence studies applied probability based sampling methods [28], [39]–[42], and only one describes the method in sufficient detail [28], but even this study is biased, because herds were selected based on proximity to a reliable laboratory and farmer cooperation. Studies of intensive farms have focused mainly on infertility or abortion outbreaks, and few cattle were sampled (Table 1). Most intensive system studies were undertaken in the West before 1986 (Figures 2A and 3), a period of intense interest in the livestock sector (Box 1 and Table 1, Row A). Since 1986, more investigations have been reported in extensive cattle systems (Table 1, Row B) and from abattoirs (Table 1, Row F). Clearly there are few good-quality data on brucellosis in Nigeria, and discussion must bear in mind these limitations.

### Extent to which the extensive and intensive cattle management systems are affected by brucellosis

In Nigeria, most cattle are reared extensively in the North and belong to nomadic, seminomadic or transhumant Fulani pastoralists. According to early official veterinary records, brucellosis was not regarded as a hazard in these herds [29], [43] and most studies conducted independently in the extensive and intensive systems suggest a lower prevalence in the former (Tables 1 and 3, Rows A and B; Table S4). This was the view of early investigators [13], [32]. Esuruoso wrote, “Cattle…in nomadic herds…on the move… are not likely to accumulate infection or spread it from one animal to the other as in settled herds. This factor, and the intense heat of the sun in fairly open country (Sudan Savannah zone) will provide some of the reasons for the low infection rate…in the northern herds… It would appear, therefore, that nomadic herding in Nigeria imposes a natural limit on the rate of brucellosis infection in cattle.” This observation is consistent with the low transmission deemed typical of pastoralist systems [7].

The inverse profile can be observed for studies that have looked at intensive and extensive system populations in parallel (Tables 1 and 3, Rows C and D; Table S5). A recent probability sampling study [28] (performed in Adamawa, Kaduna, and Kano, northern Nigeria), reports RBT seroprevalences of 45.1% (nomadic), 22.0% (seminomadic), 23.8% (commercial), and 15.9% (zero-grazing). Using a competitive ELISA kit as the reference, the authors assumed that 42.8% to 24.7% of these RBT results were false positives, but higher prevalence in the extensive than intensive system was also observed with the ELISA. Another recent, but more limited, work reported higher (but not statistically significant) numbers of RBT positives in extensively than in intensively managed herds (11.6% versus 3.1%, respectively) in Plateau State (North Central Nigeria) [42]. These results suggest that brucellosis prevalence has been on the increase in extensive systems over time [28]. However, in a recent cross-sectional survey using RBT standardised according to OIE criteria, seminomadic Fulani cattle (n = 2000) showed less than 1% individual seroprevalence in the Kachia Grazing Reserve (Kaduna) (ICONZ, 2013, www.iconzafrica.org). The reasons for the differences between this and earlier work are unclear. Although intensification provides opportunities for better control measures, their implementation cannot be taken for granted because this requires adequate infrastructure and training and, indeed, the risks of transmission are greatly increased [1], [6], [7]. None of these recent studies describe control measures in intensively managed herds that could account for the lower prevalence reported. On the other hand, at least in the Kachia Grazing Reserve, Fulani have intuitive disease-reducing management approaches (e.g., rapidly selling or slaughtering animals that abort and those with poor fertility or low milk yields), and low reproductive rates reduce transmission [7]. As discussed below, these aspects of brucellosis epidemiology are not trivial, and further studies are necessary to confirm whether there is an increase of brucellosis in extensively managed herds and its distribution across the country. Unfortunately, the gap in information between the early 1980s and late 1990s precludes any possibility of doing this with the data available (Figure 2A).

### Extensive nomadic herds as reservoirs of disease

Brucellosis transmission is generally lower in pastoralist systems because of low reproductive rates, animal movements and environmental circumstances [7]. However, brucellosis transmission could increase as a result of the settling of migratory herds and emerge from increased contacts between these herds and unprotected intensive commercial or settled semi-intensive herds. This possibility has seldom been investigated in sub-Saharan Africa. One article provides evidence of this kind of transmission and of its dramatic impact on susceptible populations in the 1970s [23]. In a large brucellosis outbreak in Ibapara, out of ten governments, three private settled, and 12 Fulani herds tested, 11 herds were found to be positive using a combination of the MRT and Card Test. All 11 positive herds belonged to Fulani pastoralists, “nomadic herdsmen that move only within the district, and within few kilometres from previous settlements.” The outbreak coincided with the Sahelian drought that saw a general reduction in the cattle population of Nigeria and prompted an influx and settling of nomadic herds in Ibapara. The outcome was a widespread epidemic of bovine brucellosis with a severe increase in human cases. Fulani herdsmen complained of being unwell and unable to look after their cattle, and 51.5% of herdsmen, 23.5% of abattoir workers, and 3.1% of high school students were serologically positive with the Card Test. Calf losses were reported, resulting in a shortage of meat and protein undernutrition in the local populace.

### Brucellosis in small ruminants

Small ruminants represent a major source of meat in Nigeria and are often reared alongside cattle. Their distribution is not known with certainty; Falade *et al.* [44] cite early sources, according to which 70% of goats were in the North, 20% in the East and 10% in the West, and about 60% of rural households in the northern, 50% in the eastern and 40% in the western states kept goats.15% of sheep and goats were reared under nomadic conditions at the end of the 20th century [22].

Bacteriological evidence for *Brucella* in small ruminants is scarce (Figure 3; Table S15). An early study claimed the isolation of *B. abortus* in sheep and goats, but the methodology used in species identification is unclear [45]. *B. melitensis* biovar 1 (22 strains) and *B. abortus* biovar 1 (8 strains) were isolated from goats in western Nigeria [46]. However, the reported biochemical characteristics of the *B. melitensis* strains are atypical. *B. melitensis* was recently described in sheep and goats in northern Nigeria but the ten strains were not definitively typed [24]. A study in Bauchi (central Nigeria) clearly demonstrated *B. abortus* but not *B. melitensis* in sheep [33]. Interestingly, seven *B. abortus* strains were isolated from sheep reared in contact with infected cattle [47]. Although *B. abortus* preferentially infects cattle, it is known to persist in sheep [48] and the significance of *B. abortus* infection in small ruminants in the mixed breeding systems of sub-Saharan Africa requires further investigation.

There are fewer and more limited serological studies in small ruminants than in cattle (Figure 2B; Tables 2 and 4; Tables S10, S11, S12, S13, S14). Significant misuse of tests were application of MRT (not useful in small ruminants [49]) in four studies and interpretation that animals were infected by *B. melitensis* based on a comparison of titres to *B. abortus* and *B. melitensis* antigens [50]–[52], a discrimination that is not possible by serology and indicates inadequate antigen standardization.

Studies in intensive or semi-intensive systems are not only scarce but also biased because most investigations focused on cattle abortions with simultaneous sampling of small ruminants (compare references in Tables 1 and 2 and Tables S3 and S10). In fact, contagion from cattle was often considered the origin of infection. Only one study was performed on intensively or semi-intensively raised small ruminants in the West [44]; the others for this region consisted of abattoir surveys (Tables 2 and 4). Studies in extensive systems were all undertaken in the North (Rows B and D in Tables 2 and 4; Table S11 and S12); hence, the epidemiology in sedentary and nomadic flocks in other regions is unknown. Although values broadly suggest that brucellosis prevalence is higher in intensive than extensive systems for small ruminants (Tables 2 and 4, Rows A, B, C, and D, Tables S10, S11, S12) these trends have to be interpreted with caution.

According to two studies performed in the 1960s, small ruminant brucellosis was not a problem on government farms, but most surveys were undertaken in the cattle-dominated North; hence, no information was available for other regions (Figure 3B) [53], [54]. Fifteen years later, one study in northern Nigeria later found significant rates of infection (13.8% and 15.1% averages for sheep and goats, respectively) [55]. This same study reported rates of infection in institutional (i.e., intensive) flocks about four times higher than in local (extensive) flocks for both sheep and goats (Table 2), and attributed the difference to an increased transmission caused by intensification [55]. A recent study [56] found overall prevalence values of 9.3% for sheep and 10.1% for goats, which are comparable to the values found 30 years previously [55], but husbandry-specific values were not obtained.

Ten studies have investigated sheep and goats for brucellosis in trade settings (Table 2, Row F; Table S14), and while values do not reflect the situation at farm level, they confirm the presence of brucellosis in small ruminants in the North. Two abattoirs studies in the West found low prevalence values (0.3%–0.9% and 0% for goat and sheep, respectively) [57], [58], but since animals come mostly from other parts of Nigeria, the situation in the West remains unknown.

### Brucellosis in other animals

*B. abortus* has been isolated from horses [33], [59], and antibodies have been reported in donkeys [60], dogs [61]–[63], and fowl [64]–[67] in Nigeria (Figure 2B). However, the role of these nonruminant species in disease transmission has never been satisfactorily proven [68] and, as they are unable to act as reservoirs, once brucellosis is eradicated in domestic ruminants, they are considered as spillover hosts or sentinels.

Camels are distributed along the northern borders of Nigeria, and nomadism is common, often across borders. At the turn of the 20th century, estimated numbers of camels in Nigeria varied from 90,000 [14] to 25,000, substantially greater than an estimate of 18,000 in 1978 [69]. Both *B. abortus* and *B. melitensis* can infect camels, but *Brucella* has never been isolated from these animals in Nigeria [70]–[72]. Serological studies are particularly difficult to interpret because brucellosis tests have not been properly evaluated in these animals [73]. Abattoir studies in northern Nigeria reported 1.3%–14.8% seropositivity using SAT [14], [69], [74], [75] in camels from Nigeria and Chad, Niger, and Cameroon (Figure 3C). In Borno State, two MRT and RBT studies of range camels reported positive animals [70], [75]. However, the MRT has been proven useful only in cattle [49], and the RBT is dependent on the effect of acidic pH on ruminant IgG and IgM [76], [77]. Since camelids and ruminants differ markedly in immunoglobulin repertoire and structure [78], RBT results should be interpreted with caution. Camels are herded with sheep and goats and, to a lesser extent, cattle [69], and their role in the epidemiology of brucellosis in Nigeria is unclear.

Pigs represent approximately 4.5% of the meat market in Nigeria [79]. An early study claimed isolation of *B. suis* from animals positive in SAT [80] but a small-scale bacteriological study failed to isolate *Brucella* [33]. An investigation in government farms during a cattle abortion outbreak [53], a study in intensive and semi-intensive farms in the South [79], and an abattoir study in the West [58] found no or very few RBT positive animals. In contrast, a recent abattoir study in Central Nigeria reported 30% of 281 pigs RBT positive (Figure 3C) [81]. In the absence of bacteriological evidence or protein-based tests, these data have to be interpreted with caution, because pigs are prone to false positive serological reactions with RBT, CFT, and ELISA [82].

### Control of animal brucellosis

Brucellosis control was initiated in colonial Nigeria in 1917; vaccination was applied to address widespread bovine abortions in government-owned farms and local production of a liquid S19 vaccine started at this time. A test and slaughter policy was also implemented [83], and its failure was attributed to a lack of rigor in implementation [28]. Production of lyophilised S19 started in 1950 [12], and by 1951, brucellosis eradication and control programmes succeeded in establishing brucellosis-free stock and reducing overall prevalence to less than 5% on government farms [28]. Efforts waned and vaccine production discontinued in 1954 [12] and today there is no government policy for brucellosis control in Nigeria. Nevertheless, local researchers estimated that brucellosis caused approximately 20% financial losses in traditional systems of cattle production in one Nigerian grazing reserve [84] and concluded that, as the nomads settle in these reserves, hygienic measures and brucellosis vaccination are profitable and should be implemented [85]. A recent study identified brucellosis and milk loss as the greatest components of the direct economic losses associated with reproductive disorders in settled herds in Zaria, Nigeria [86].

### Human brucellosis

The first cases of human brucellosis confirmed by laboratory tests were reported in Nigeria in 1941 [87] and 1962 [88], and even during this period, underdetection was suspected [89]. A decade later, few laboratories could perform these tests and this, combined with low suspicion, was again thought to lead to underdetection [90]. This review shows that these circumstances have not changed.

Human seroprevalence data are summarized in Table 5, and Figure 3D shows the geographical location of studies. Although they strongly suggest the importance of the human disease, exact figures cannot be derived from most surveys. The studies based solely on RBT confirm exposure to *Brucella* of butchers, abattoir workers, and herdsmen. However, they do not necessarily represent the proportion of true disease, because a positive RBT result can be caused by contact or infection and needs to be interpreted according to the clinical picture [76]. Several studies complemented RBT with SAT and 2-mercaptoethanol tests, both of which detect only agglutinating antibodies; since these antibodies disappear in long-standing cases, the data only reflect recent infections. Moreover, SAT diagnostic titre varies from 50 to 200 international units (the diagnostic titre most often used in Nigeria was of 100 international units) depending on the origin (urban or rural and endemic or non-endemic areas) and exposure of the patient [76]. Complementary tests that detect non-agglutinating antibodies (competitive ELISA, Coombs, and CFT) were implemented in only two studies, one using competitive ELISA whose diagnostic cut-off for human brucellosis is unknown [76].

**Table 5:** Summary of brucellosis studies in humans in Nigeria.

	Region	Diagnostic test (cut-off)	Complementary tests	% Prevalence (n)	Refs.
**Occupationally exposed**					
Abattoir workers	West	SAT (100 iu)	2-ME	39 (170)	[25]
	West	RBT		24 (51)	[23]
	North	RBT	SAT	0 (40)	[123]
	South	SAT (NS)		27 (164)	[125]
Butchers & abattoir workers	West	RBT		64 (11)	[58]
Butchers	West	SAT (100 iu)	2-ME	21 (38)	[25]
	West	SAT (100 iu)	2-ME	16 (51)	[25]
	North	RBT	SAT	5 (101)	[113]
Herdsmen	West	SAT (100 iu)	2-ME	74 (104)	[25]
	West	SAT (100 iu)	2-ME	12 (99)	[25]
	West	SAT (100 iu)	2-ME	5 (44)	[25]
	North	SAT (100 iu)		70 (71)	[126]
	West	RBT	2-ME	51 (173)	[23]
	West	SAT (100 iu)	2-ME	7 (20)	[102]
	West	RBT		0 (10)	[58]
	North	RBT	SAT, c-ELISA	7 (28)	[101]
Veterinary workers	West	SAT (100 iu)	2-ME	5 (44)	[25]
	South	SAT (NS)		16 (86)	[125]
Cattle control post workers	West	SAT (100 iu)	2-ME	21 (18)	[25]
Agricultural college students	West	SAT (100 iu)	2-ME	12 (300)	[102]
**Hospital studies**					
*Febrile individuals*					
Students	North	RBT	SAT	8 (122)	[127]
Civil servants	North	RBT	SAT	4 (100)	[127]
Traders	North	RBT	SAT	2 (53)	[127]
Housewives	North	RBT	SAT	2 (62)	[127]
Crop farmers	North	RBT	SAT	0 (6)	[127]
Health workers	North	RBT	SAT	0 (10)	[127]
Children (1–15 years)	North	RBT	SAT	10 (93)	[127]
Village farmers	North	RBT	SAT	6 (114)	[91]
Traders and breeders	North	RBT	SAT	34 (62)	[91]
Abattoir workers, butchers	North	RBT	SAT	44 (32)	[91]
Civil servants	North	RBT	SAT	4 (634)	[91]
Others	North	RBT	SAT	6 (198)	[91]
*Not specified*					
Patients	West	SAT (50 iu)	RBT, Coombs, CFT	6 (738)	[128]
Patients and personnel	West	SAT		9 (176)	[129]
Patients and personnel	North	RBT	SAT	0 (64)	[123]
Personnel	North	RBT	SAT	0 (90)	[123]
Blood donors, ante-natal women, male patients	West	SAT (100 iu)	2-ME	11 (1192)	[25], [130]
Blood donors	West	SAT (100 iu)	2-ME	21(178)	[25]
Blood donors	South	SAT (NS)		12 (50)	[125]
**Others**					
High school students	West	RBT		3(65)	[23]

There are no reports of *Brucella* isolation from human cases, and it is not known to what extent human brucellosis in Nigeria is caused by *B. abortus* or *B. melitensis*. Interpretation of human infection caused by *B. melitensis* or *B. abortus* on the basis of different titres with *B. melitensis* and *B. abortus* antigens is deceptive [91]. Misdiagnosis may be frequent; one abattoir study found that RBT positive individuals often complained of frequent treatments for malaria without showing improvement, while others complained of joint pain and general weakness [58].

### Conclusion: Lessons from Nigeria

This review has identified major gaps in epidemiological data, diagnostics, and control, and misconceptions surrounding brucellosis. After 100 years, we know surprisingly little on the disease agent in Nigeria, and good-quality information—essential for evaluation of zoonotic potential and for establishment of control measures—is still lacking. Bacteriological studies are necessary to clarify the picture of both animal and human brucellosis. Preliminary evidence suggests that *B. abortus* biovar 3a is dominant or restricted to Africa, but little is known about its virulence and other biological properties. Also, the existence and distribution of *B. melitensis* and *B. suis* needs to be clarified. Likewise, a judicious choice of serological tests validated under local conditions and an understanding of their value in different contexts is key, as is implementation of clinical protocols and simple affordable tests for routine diagnosis in humans. Most sophisticated serodiagnostic tests were developed in high-income countries many years after brucellosis was eradicated, and these tests are better suited to epidemiological surveillance in well-equipped laboratories. Capacity building is a clear need, and the establishment of a reference laboratory for both human and animal brucellosis in sub-Saharan Africa would be a great asset.

The outbreak investigated by Alausa over 30 years ago [23] may be highly significant, because it shows the dramatic effect of the influx and settling of infected nomadic herds in areas where no control measures are implemented. This can happen in contemporary Nigeria where rural–urban migration, changing trends in livestock management and increased intensification could re-create the conditions for emergence of disease [6]. Climate change and desertification of the Sahel may also be an important driver for emergence, as it accounts in part for rural–urban migration [9] and is predicted to cause a reduction in the number of crop farmers in favour of livestock keepers [10]. Settling of nomadic Fulani in peri-urban areas and grazing reserves may be advantageous politically and economically, opening market chains for dairy products, offering formalised access to education and healthcare services, and avoiding disputes over land-use and clashes with crop farmers [92]. The emergence of brucellosis could, in these circumstances, have far-reaching social and political implications [84], [93], [94].

Prophylaxis and control of brucellosis requires contextual adaptation. Most evidence suggests differences in epidemiology between extensive livestock production systems and more intensive systems worldwide [1], [7]. This could apply to past situations in Nigeria, but we do not have a clear picture of the present status of the disease. An understanding of the dynamics of brucellosis in nomadic pastoralist systems and at the interface with settled populations is critical. Mass-vaccination approaches may be difficult to implement in extensively managed animals in Nigeria, but it is essential they be applied in the intensive and commercial systems. At a time when cost-effectiveness needs to be demonstrated, brucellosis control measures should be focused on settled populations that are at risk. This appeals to policy-makers, as settled populations are accessible and more amenable to mass-vaccination campaigns than nomadic pastoralist communities. Moreover, since differentiation of infected and vaccinated animals is not critical initially, the most effective vaccines (S19 in cattle and, if necessary, Rev1 in small ruminants [95]) should be used.

Nomadic pastoralism could offer a well-adapted management system for disease mitigation in Nigeria; if the disease exists at low levels, animals exhibit a low overall frequency of abortion and there are few opportunities for disease transmission. One Health and Eco Health approaches to disease reduction and prevention are particularly relevant in pastoralist communities, considering that pastoralism and transhumance is a desirable livelihood strategy in Nigeria [96].

Currently there is no coordinated policy for brucellosis in Nigeria. An assessment of the direct and indirect impact of brucellosis on these communities leading to culturally appropriate and locally adapted control options is overdue. There is a need to undertake a countrywide, evidence-based, and multidisciplinary study of brucellosis in the different livestock production systems of Nigeria to determine the extent, potential impact, and origin of brucellosis and to propose control template strategies of proven efficacy.

**Key Learning Points**

- Despite imperfect evidence, an exhaustive review of studies in Nigeria suggests that brucellosis persists at low endemic levels in nomadic pastoralist systems. The settling of nomadic or transhumant pastoralist populations and intensification in livestock management, may favour disease transmission and conditions for brucellosis outbreaks.
- There is an urgent need to study the dynamics of the disease at the interface between extensive pastoralist and intensive or settled livestock systems and to implement brucellosis control measures adapted to each of these situations.
- The few attempts to implement a vaccination plus test and slaughter strategy in cattle show that this approach was not sustainable.
- The role of small ruminants and camels in the epidemiology of brucellosis in Nigeria remains unknown because of insufficient bacteriological investigations and, for camels, properly validated serological tests.
- The extent of the public health impact of brucellosis is largely unknown, and bacteriological studies to characterise the *Brucella* species infecting humans are lacking.
- There is an imperfect understanding of the animal and human disease and of the value of the different diagnostic tests in different epidemiological contexts. One Health courses for veterinarians, medical doctors, and diagnostic laboratory personnel are necessary.

**Top Five Papers in the Field**

1. Ajogi I, Akinwumi JA, Esuruoso GO, Lamorde AG (1998) Settling the nomads in Wase-Zange grazing reserves in the Sudan Savannah zone of Nigeria III. Estimated financial losses due to bovine brucellosis. Nigerian Vet J 19: 86–94.
2. Alausa OK (1979) The investigation and control of a large-scale community outbreak of brucellosis in Nigeria. Public Health 93: 185–193.
3. Esuruoso GO (1974) Bovine brucellosis in Nigeria. Vet Rec 95: 54–58.
4. Grace D, Mutua F, Ochungo P, Kruska R, Jones K (2012) Mapping of poverty and likely zoonoses hotspots. Zoonoses Project 4. Report to the UK Department for International Development. Nairobi, Kenya: ILRI. Available: http://cgspace.cgiar.org/handle/10568/21161. Accessed 27 June 2014.
5. Waters-Bayer AN, Bayer W (1994) Coming to Terms. Interactions between Immigrant Fulani Cattle-Keepers and Indigenous Farmers in Nigeria's Subhumid Zone. Cahiers d'Études Africaines 34: 213–229. doi:10.3406/cea.1994.2048

## Supporting Information

Table S1 Rejected brucellosis serology studies in cattle.
(DOCX)

Table S2 Rejected brucellosis serology studies in sheep and goats.
(DOCX)

Table S3 Brucellosis serology studies in cattle reared under intensive livestock systems.
(DOCX)

Table S4 Brucellosis serology studies in cattle reared under extensive livestock systems.
(DOCX)

Table S5 Brucellosis serology studies in cattle undertaken in extensive and intensive livestock systems in parallel.
(DOCX)

Table S6 Brucellosis serology studies in cattle reared under non-specified livestock systems.
(DOCX)

Table S7 Brucellosis abattoir serology studies in cattle.
(DOCX)

Table S8 Brucellosis milk market milk serology studies in cattle.
(DOCX)

Table S9 Brucellosis bacteriology studies in cattle.
(DOCX)

Table S10 Brucellosis serology studies in sheep and goats reared under intensive livestock systems.
(DOCX)

Table S11 Brucellosis serology studies in sheep and goats reared under extensive livestock systems.
(DOCX)

Table S12 Brucellosis serology studies in sheep and goats undertaken in extensive and intensive livestock systems in parallel.
(DOCX)

Table S13 Brucellosis serology studies in sheep and goats under non-specified livestock systems.
(DOCX)

Table S14 Brucellosis abattoir serology studies in sheep and goats.
(DOCX)

Table S15 Brucellosis bacteriology studies in sheep and goats.
(DOCX)
