# Supplementary material for: Brucellosis as an Emerging Threat in Developing Economies: Lessons from Nigeria
Source: PLoS Negl Trop Dis. 2014 Jul 24;8(7):e3008. doi: 10.1371/journal.pntd.0003008 (PMC4109902; doi:10.1371/journal.pntd.0003008)
Supplement: Table S1 — Rejected brucellosis serology studies in cattle. (DOCX) [file pntd.0003008.s001.docx]

| **Reference** | **Study description** | **Study location** | **Region** | **Diagnostic test** | **Period of**  **sampling^[[1]](#footnote-1)^** | **n** | **Prev.** | **Reason for rejection** |
| --- | --- | --- | --- | --- | --- | --- | --- | --- |
| Mai et al., 2013 | Risk factor analysis for seroposivity | Adamawa State  Kaduna State  Kano State | North | NA | 2012 | NA | NA | Prevalence data published in Mai et al., 2012 |
| Mailafia et al., 2010 | Retrospective study of veterinary case reports | Abuja | North | Clinical diagnosis | 1999-2000 | NA | 3 cases | Brucellosis cannot be diagnosed based on clinical suspicion |
| Antiabong et al., 2009 | Molecular characterisation | NS | North | Broth culture & PCR | 2009 | NA | NA | No field strains characterised, proof of concept study for combination of broth culture and PCR methods |
| Ishola & Ogundipe, 2001 | Abattoir | Ibadan | West | RBT | 2009 | 398 | 6.28 | Same study and data as Ishola & Ogundipe, 2000 |

NS- not specified, NA- not applicable, PCR- polymerase chain reaction, RBT- rose Bengal test, Prev.- prevalence

1. When period of study not specified, year of publication used [↑](#footnote-ref-1)
