# Supplementary material for: Brucellosis as an Emerging Threat in Developing Economies: Lessons from Nigeria
Source: PLoS Negl Trop Dis. 2014 Jul 24;8(7):e3008. doi: 10.1371/journal.pntd.0003008 (PMC4109902; doi:10.1371/journal.pntd.0003008)
Supplement: Table S2 — Rejected brucellosis serology studies in sheep and goats. (DOCX) [file pntd.0003008.s002.docx]

| **Reference** | **Study description** | **Study location** | **Region** | **Diagnostic test** | **Period of**  **sampling^[[1]](#footnote-1)^** | **n** | **Prev.** | **Reason for rejection** |
| --- | --- | --- | --- | --- | --- | --- | --- | --- |
| Mailafia et al., 2011 | 10 year retrospective study on case reports at Ministry vet clinic | Abuja | North | None (case reports) | 1999-2008 | NA | 10 cases (G)  10 cases (S) |  |
| Bukar-Kolo et al., 2007 | Isolation of bacteria from anterior vagina of does | Maiduguri | North | Bacteriology | 2007 | 103 (G) | 0 | Did not use selective media or adequate incubation conditions for isolation of *Brucella* |
| Falade, 1981 | Rev 1 experimental vaccination and post-vaccination serological monitoring | Ibadan | West | RBT  SAT  CFT  Rivanol | 1981 | NA (G) | NA | No data on prevalence due to natural infections |
| Okoh, 1980b | Abortion investigation in LIBC | Rano, Kano State | North | SAT | 1977 | 372 (S) | 14.5 | Same study as Okoh, 1980a |
| Falade, 1978 | Comparison of 3 serological tests | Northern, Southern and Western States | North  South  West | RBT  SAT  MRT | 1978 | 2550 (G)  590 (G) | 5.95  4.27  48.9 | Same animals as Falade et al. (1974) |

NS- not specified, NA- not applicable, SAT-serum agglutination test, RBT- rose Bengal test, CFT- complement fixation test, MRT- milk ring test, Prev.- prevalence, S sheep, G- goat, LIBC- livestock breeding and investigation centre

1. When period of study not specified, year of publication used [↑](#footnote-ref-1)
