# Supplementary material for: Brucellosis as an Emerging Threat in Developing Economies: Lessons from Nigeria
Source: PLoS Negl Trop Dis. 2014 Jul 24;8(7):e3008. doi: 10.1371/journal.pntd.0003008 (PMC4109902; doi:10.1371/journal.pntd.0003008)
Supplement: Table S4 — Brucellosis serology studies in cattle reared under extensive livestock systems. (DOCX) [file pntd.0003008.s004.docx]

| **Reference** | **Population** | **Sampling**  **method** | **Sampling**  **approach** | **Bias**  **(gaps in method description)** | **Diagnostic test^[[1]](#footnote-1)^**  **(cut-off)** | **Period of**  **sampling^[[2]](#footnote-2)^** | **Region** | **Location^[[3]](#footnote-3)^** | **Sample size**  **(no. herds)** | **Prevalence**  **(herd prev.)**  **%** | **Comments** |
| --- | --- | --- | --- | --- | --- | --- | --- | --- | --- | --- | --- |
| Mbuk et al., 2011 | Migratory Fulani herds | NPS | 15 LGAs selected randomly, 10% cattle sampled in selected herds | Animals with brucellosis clinical signs preferentially sampled  (Method for selection of herds not described) | RBT | 2011 | North | Kaduna State  *Birnin Gwari*  *Chikun*  *Kaduna North*  *Kachia*  *Lere*  *Kaduna South*  *Zango-Kataf*  *Kaura*  *Jemma’a*  *Jaba*  *Giwa*  *Zaria*  *Soba*  *Kudan*  *Igabi* | 2799 (93)  *199*  *178*  *157*  *176*  *184*  *158*  *205*  *201*  *201*  *192*  *193*  *193*  *181*  *188*  *183* | 7.1 (NS)  *5.5*  *3.4*  *2.5*  *3.4*  *1.6*  *5.1*  *16.1*  *9.5*  *14.4*  *8.8*  *4.7*  *4.7*  *8.8*  *7.4*  *8.7* |  |
| Ocholi et al., 1996 | Settled Fulani herds | NPS?^[[4]](#footnote-4)^ | Randomly selected herds | (Method not well characterised) | RBT | 1996 | North | Kaduna State | 762 (40) | 2.1 (NS) |  |
| Farouk et al., 2013 | Pastoralist herds | PS?^[[5]](#footnote-5)^ | 10 herds selected by balloting per each senatorial district | (Method not well characterised) | MRT | 2013 | West | Jigawa State | 260 (30) | 3.5 (NS) |  |
| Bertu et al., 2010 | Fulani herds | NPS? | Random sampling | (Method not well characterised) | MRT | 2005 | North | Jos | 100 (NS) | 15 (NS) |  |
| Pullan, 1980 | Settled Fulani and indigene herds | NPS | Purposive sampling | Herds purposively selected according to non-migratory behaviour and to represent diversity of management systems | MRT/RBT | 1975-1976 | North | Plateau State  *Jos Plateau* | 1053 (8) | 5.1 (12.5) | 1 herd found MRT positive and 24% of 54 animals in herd RBT positive. |

NS- not specified, NPS- non-probability sampling, PS- probability sampling, RPT- rapid plate test, SAT- serum agglutination test, RBT- rose Bengal test, MRT- milk ring test, no. - number

1. One test seroprevalence value per study reported in this preferential test order: RBT, CT, CFT, RPT, SAT, MRT. For studies that do not report parallel test results, seroprevalence value obtained with tests used in series reported (see text). [↑](#footnote-ref-1)
2. When period of study not specified, year of publication used. [↑](#footnote-ref-2)
3. If the samples originate from more than one area, individual prevalence for each area is reported if the information is available. [↑](#footnote-ref-3)
4. NPS? Denotes that the method is not described but that non-probability sampling in most likelihood applies. [↑](#footnote-ref-4)
5. PS? Denotes that the sampling method is not well described but that probability sampling in most likelihood applies. [↑](#footnote-ref-5)
