# Supplementary material for: Brucellosis as an Emerging Threat in Developing Economies: Lessons from Nigeria
Source: PLoS Negl Trop Dis. 2014 Jul 24;8(7):e3008. doi: 10.1371/journal.pntd.0003008 (PMC4109902; doi:10.1371/journal.pntd.0003008)
Supplement: Table S5 — Brucellosis serology studies in cattle undertaken in extensive and intensive livestock systems in parallel. (DOCX) [file pntd.0003008.s005.docx]

| **Reference** | **Population** | **Sampling method** | **Sampling approach** | **Bias**  **(gaps in method description)** | **Diagnostic test^[[1]](#footnote-1)^**  **(cut-off)** | **Period of**  **sampling^[[2]](#footnote-2)^** | **Region** | **Location^[[3]](#footnote-3)^** | **Livestock system** | **Sample size**  **(no. herds)** | **Prevalence**  **(herd prev.) %** | **Comments** |
| --- | --- | --- | --- | --- | --- | --- | --- | --- | --- | --- | --- | --- |
| Maurice et al., 2013 | Pastoralist herds  Sedentary herds | PS? | Cross-sectional survey | Sampling frame or method not described | RBT | 2013 | North | Plateau State | EXT  INT | 206 (NS)  64 (NS) | 11.6 (NS)  3.1 (NS) |  |
| Cadmus et al., 2013 | Nomadic Fulani herds  Sedentary herds | PS?^[[4]](#footnote-4)^ | Every 3^rd^ herd selected, 5% of herd sampled | Small sample size  (Sampling frame not described) | RBT | 2013 | West | Ogun State,  Yewa Division | EXT  INT | 64 (NS)  88 (NS) | 4.7 (NS)  7.9 (NS) |  |
| Mai et al., 2012 | Pastoralist and agropastoralist Fulani herds; commercial paddocked and zero-grazing herds | PS | Single stage cluster sampling | Herd selection based on proximity to reliable lab and farmer cooperation | RBT/  C-ELISA  (60% OD)^6^ | 2012 | North | Adamawa, Kaduna  Kano States | COM  EXT(Past.)  EXT (Agro.)  INT (Comm.)  INT (0-graz) | 4745 (271)  1244 (NS)  2758 (NS)  642 (NS)  101 (NS) | 36.6 (84.9)  45.1 (NS)  22.0 (NS)  15.9 (NS)  23.8 (NS) | Non-adjusted prev.^6^:  40.8%  19.5%  14.1%  21.1% |
| Nuru & Dennis, 1975 | Fulani herds  Government ranches | NPS?^[[5]](#footnote-5)^ | NS | Cows observed to have clinical signs of brucellosis sampled  (Method characterised in thesis which was not retrievable) | RPT  (1:50) | 1972-1974 | North | Overall (Fulani)  NE State  NW State  N Central State  Benue/Plateau State  Kano State  Kwara State  Overall (Gov) | EXT  EXT  EXT  EXT  EXT  EXT  EXT  INT | 805 (255)  177 (NS)  151 (NS)  149 (NS)  159 (NS)  29 (NS)  140 (NS)  227 (11) | 8.8 (NS)  6.7 (NS)  5.3 (NS)  2.0 (NS)  3.8 (NS)  0 (NS)  30 (NS)  9.7 (NS) | Same Fulani herd prevalence obtained as for Banerjee & Bhatty (1970) study |
| Banerjee & Bhatty, 1970 | Fulani herds  Government LIBC herds | NPS | Convenience sampling | Sera sent to NVRI for various diagnostic purposes, especially for CBPP diagnosis | RPT  (1:50) | 1970 | North | NW State, N Central State, NE State, Kano State, Benue/Plateau State | EXT  INT | 1,650 (NS)  2350 (NS) | 8.8 (NS)  3.7 (NS) | Vaccination campaign with S19 undertaken in government herds |
| Esuruoso, 1974a | Fulani herds, government herds | NPS | Purposive sampling to investigate abortion/fertility problems | Herd selection based on request for investigation from Chief Veterinary Officer of each State | SAT(160iu)/CFT (1:40) | 1968-1974 | North  West | Kano State  *Kano city*  *Kadawa**  *Kano dairy**  *Gayo*  NW State  *Minna**  *Mokwa*  Kwara State  *Ilorin*  *Emiryard*  *Ajara*  *New Bussa*  Benue/Plateau State  *Vom**  *Kuru*  West State  *Unife* | COM  EXT  INT*  INT*  INT  COM  INT*  INT  COM  INT  EXT  EXT  EXT  COM  INT*  INT  COM  INT | 53 (4)  *5 (1)*  *10 (1)**  *11 (1)**  *27 (1)*  117 (2)  *78 (1)**  *39 (1)*  113 (4)  *62 (1)*  *30 (1)*  *14 (1)*  *7 (1)*  52 (2)  *37 (1)**  *15 (1)*  33 (1)  *33 (1)* | 9.4 (75)  *0 (0)*  *20 (100)**  *18 (100)**  *3.7 (100)*  11.1 (100)  *11.5 (100)**  *10.3 (100)*  3.5 (50)  *5 (100)*  *3.3 (100)*  *0 (0)*  *0 (0)*  3.8 (50)  *0 (0)**  *13.3 (100)*  33.3 (100)  *33.3 (100)** | *Vaccinated herds  Management system for non-Fulani herds not specified but can assume that intensive management probably applies.  Samples considered positive when they were positive for SAT confirmed by CFT for suspicious samples. |

NS- not specified, NPS- non-probability sampling, PS- probability sampling, RPT- rapid plate test, SAT- serum agglutination test, RBT- rose Bengal test, MRT- milk ring test, CT- card test, no. – number, EXT- extensive, INT- intensive, COM- combined, LIBC- livestock investigation and breeding centre, Past.- patoralist, Agro.- agropastoralist, Comm.- commercial, 0-graz.- zero-grazing, Gov.- government.

1. One test seroprevalence value per study reported in this preferential test order: RBT, CT, CFT, RPT, SAT, MRT. For studies that do not report parallel test results, seroprevalence value obtained with tests used in series reported (see text). [↑](#footnote-ref-1)
2. When period of study not specified, year of publication used [↑](#footnote-ref-2)
3. If the samples originate from more than one area, individual prevalence for each area is reported, if not, the overall state/region prevalence is reported [↑](#footnote-ref-3)
4. PS? Denotes that the sampling method is not well described but that probability sampling in most likelihood applies [↑](#footnote-ref-4)
5. NPS? Denotes that the method is not described but that non-probability sampling in most likelihood applies

   ^6^ Individual prevalences adjusted for sensitivity and specificity of serial testing system (samples positive or inconclusive with RBT confirmed with C-ELISA). Non-adjusted prevalence calculated as per Mai *et al.* 2012 (apparent prevalence = [true prevalence (0.879+0.998-1)] +1 – 0.998]; 0.998= specificity of RBT*ELISA in test series; 0.879=sensitivity of test series, AP=TP[Se+Sp-1]+1-Sp). [↑](#footnote-ref-5)
