# Supplementary material for: Brucellosis as an Emerging Threat in Developing Economies: Lessons from Nigeria
Source: PLoS Negl Trop Dis. 2014 Jul 24;8(7):e3008. doi: 10.1371/journal.pntd.0003008 (PMC4109902; doi:10.1371/journal.pntd.0003008)
Supplement: Table S6 — Brucellosis serology studies in cattle reared under non-specified livestock systems. (DOCX) [file pntd.0003008.s006.docx]

| **Reference** | **Population** | **Sampling method** | **Sampling approach** | **Bias**  **(gap in method description)** | **Diagnostic test^^[[1]](#footnote-1)^^**  **(cut-off)** | **Period of**  **sampling^[[2]](#footnote-2)^** | **Region** | **Location^^[[3]](#footnote-3)^^** | **Sample size**  **(no.herds)** | **Prevalence**  **(herd prev.) %** | **Comments** |
| --- | --- | --- | --- | --- | --- | --- | --- | --- | --- | --- | --- |
| Bertu et al., 2012 | NS | NPS | Convenience sampling | Samples submitted to NVRI on suspicion of brucellosis | RBT | 2004-2009 | North | Overall  Abuja State  Adamawa State  Bauchi State  Kano State  Kano State  Nassawara State  Niger State  Plateau State  Taraba State  Taraba State | 2276 (NS)  80 (NS)  74 (NS)  447 (NS)  51 (NS)  977 (93)  16 (NS)  417 (NS)  89 (NS)  60 (NS)  65 (9) | 12.7 (NS)  3.8 (NS)  45.3 (NS)  3.0 (NS)  0 (NS)  1.8 (14)  50.0 (NS)  46.4 (NS)  12.9 (NS)  8.3 (NS)  6.2 (22.2) |  |
| Junaidu et al., 2011 | Breeding herds | PS?^[[4]](#footnote-4)^ | Multistage cluster? | (Method poorly characterised) | RBT | 2008 | North | Sokoto State  *Isa*  *Sokoto*  *Gwadabawa*  *Tambuwal* | 1557 (65)  *349 (NS)*  *444 (NS)*  *453 (NS)*  *311 (NS)* | 19.6 (NS)  *22.3 (NS)*  *20.0 (NS)*  *18.3 (NS)*  *17.6 (NS)* |  |
| Cadmus et al., 2006 | Resident farms | NPS?^[[5]](#footnote-5)^ | NS | (Method not characterised) | RBT | 2004 | West | Ibadan | 93 (7) | 0 |  |
| Alausa, 1979 | 10 government, 3 private settled, 12 nomadic Fulani herds | NPS | Outbreak investigation | (Method not characterised) | CT/  MRT | 1976 | West | Oyo State  Ibapara District | 1650 (25) | 47.4 (44) | All 11 positive herds are Fulani herds.  Positive results correspond to CT positive, MRT positive, or both. |

NS- not specified, NPS- non-probability sampling, PS- probability sampling, RBT- Rose Bengal test, CT- Card Test, MRT- milk ring test, no. - number

1. One test seroprevalence value per study reported in this preferential test order: RBT, CT, CFT, RPT, SAT, MRT. For studies that do not report parallel test results, seroprevalence value obtained with tests used in series reported (see text). [↑](#footnote-ref-1)
2. When period of study not specified, year of publication used [↑](#footnote-ref-2)
3. If the samples originate from more than one area, individual prevalence for each area is reported, if not, the overall state prevalence is reported [↑](#footnote-ref-3)
4. NPS? Denotes that the method is not described but that non-probability sampling in most likelihood applies [↑](#footnote-ref-4)
5. PS? Denotes that the sampling method is not well described but that probability sampling in most likelihood applies [↑](#footnote-ref-5)
