# Supplementary material for: Brucellosis as an Emerging Threat in Developing Economies: Lessons from Nigeria
Source: PLoS Negl Trop Dis. 2014 Jul 24;8(7):e3008. doi: 10.1371/journal.pntd.0003008 (PMC4109902; doi:10.1371/journal.pntd.0003008)
Supplement: Table S7 — Brucellosis abattoir serology studies in cattle. (DOCX) [file pntd.0003008.s007.docx]

| **Reference** | **Origin of cattle** | **Diagnostic test^^[[1]](#footnote-1)^^**  **(cut-off)** | **Period of**  **sampling^[[2]](#footnote-2)^** | **Region** | **Location**  **City (State)** | **Name of abattoir/market** | **n** | **Prev.**  **(%)** | **Comments** |
| --- | --- | --- | --- | --- | --- | --- | --- | --- | --- |
| Cadmus et al., 2013 | Ogun State, Benin, Burk. Faso, Mali, Togo, Chad, North | RBT | 2013 | West | Yewa District | Yewa Division MK | 127 | 11.0 |  |
| Cadmus et al., 2010 | Extensive herds in North | RBT | 2004-2006 | West | Ibadan | Bodija Municpal AB | 1642 | 5.9 |  |
| Cadmus et al. 2009 | North & Chad, Niger, Mali, Burk. Faso, Cameroon | RBT | 2009 | West | Lagos | Ikorodu AB  Oka-Oba AB | 126  353 | 8.7  8.5 |  |
| Cadmus et al., 2008 | NS (same as above?) | RBT | 2008 | West | Ibadan | Bodija Municpal AB | 532 | 9.8 |  |
| Cadmus et al., 2008 | NS (same as above?) | RBT | 2008 | West | Ibadan | Bodija Municpal AB | 917 | 3.5 |  |
| Cadmus et al., 2006 | North & Chad, Niger, Mali, Burkino Faso., Cameroon | RBT | 2004 | West | Ibadan | Bodija Municpal AB  Akinyele MK | 1210 | 5.8 |  |
| Bertu et al., 2012 | NS (Benue State?)  NS (Taraba State?) | RBT | 2009 | North | (Benue State)  (Taraba State) | Gboko, Katsina, Ala, Makurdi AB & MK  Ibi, Wakuri, Jalingo AB | 206  119 | 8.4  11.5 | Village abattoirs |
| Gusi et al., 2010 | NS (Plateau State?) | RBT | 2009 | North | Jos | Jos Main AB | 797 | 5.8 |  |
| Tijjani et al., 2009 | Yobe state (North?) | RBT | 2007 | East | Damaturu | Damaturu AB | 318 | 5.7 |  |
| Junaidu & Garba, 2006 | NS (Sokoto State?) | RBT | 2006 | North | Sokoto | Sokoto Metropolitan AB | 1711 | 22.4 |  |
| Ishola & Ogundipe, 2000 | North & Chad, Niger, Mali, Burk. Faso, Cameroon | RBT | 2009 | West | Ibadan | Bodija Municpal AB | 398 | 6.3 |  |
| Ajogi, 1997 | NS (North?) | RBT | 1997 | North | Jos  Zaria  Kano  Kaduna  Bauchi | NS AB  NS AB  NS AB  NS AB  NS AB | 155  48  101  264  106 | 5.2  0  0  3.8  2.6 |  |
| Ogundipe et al., 1994 | NS (same as Cadmus?) | RBT | 1992-1993 | West | Ibadan | Ibadan Municipal AB | 819 | 9.9 |  |
| Oyejide et al., 1987 | NS (same as Cadmus?) | RBT | 1987 | West | Ibadan | Ibadan Central AB | 80 | 5.0 |  |
| Chukwu, 1987 | NS (Anambra state?, North?) | RBT | 1985-1986 | South | Nsukka  Enugu | NS AB | 2050 | 6.0 |  |
| Esuruoso, 1974 | Benue-Plateau State  Benue-Plateau State  North Central State  North Western State  North & Chad, Niger | RPT (1:50) | 1972-1973 | North  West | Jos  Bukuru  Kaduna  Minna  Ibadan | NS AB  NS AB  NS AB  NS AB  NS AB | 72  12  74  12  1016 | 4.2  0  17.6  0  6.3 |  |
| Shehu et al., 1999 | NS (Bauchi State?, neighbouring countries) | SAT (NS) | 1999 | North | Bauchi | Bauchi Metropolitan AB | 1000 | 10.8 | 2 game reserves in Bauchi- wildlife reservoir? |

NS- not specified, RPT- rapid plate test, SAT- serum agglutination test, RBT- Rose Bengal test, AB- abattoir, Prev.- prevalence

1. One test seroprevalence value per study reported in this preferential test order: RBT, CT, CFT, RPT, SAT, MRT. For studies that do not report parallel test results, seroprevalence value obtained with tests used in series reported (see text). [↑](#footnote-ref-1)
2. When period of study not specified, year of publication used [↑](#footnote-ref-2)
