# Supplementary material for: Brucellosis as an Emerging Threat in Developing Economies: Lessons from Nigeria
Source: PLoS Negl Trop Dis. 2014 Jul 24;8(7):e3008. doi: 10.1371/journal.pntd.0003008 (PMC4109902; doi:10.1371/journal.pntd.0003008)
Supplement: Table S8 — Brucellosis milk market milk serology studies in cattle. (DOCX) [file pntd.0003008.s008.docx]

| **Reference** | **Origin of cattle** | **Diagnostic test^[[1]](#footnote-1)^**  **(cut-off)** | **Period of sampling^[[2]](#footnote-2)^** | **Region** | **City** | **Name of market** | **n** | **Prev.(%)** | **Comments** | |
| --- | --- | --- | --- | --- | --- | --- | --- | --- | --- | --- |
| Farouk et al., 2013 | Jigawa State | MRT | 2004-2006 | West | Ibadan | Babura, Taura, Ringim,Maigatari, Garki | 310 | 7.4 |  |  |
| Bertu et al., 2010 | Jos area, Fulani herds | MRT | 2005 | North | Jos | Informal milk sellers in Jos metropolis | 100 | 12 |  |  |

MRT- milk ring test, Prev.- prevalence

1. One test seroprevalence value per study reported in this preferential test order: RBT, CT, CFT, RPT, SAT, MRT. For studies that do not report parallel test results, seroprevalence value obtained with tests used in series reported (see text). [↑](#footnote-ref-1)
2. When period of study not specified, year of publication used [↑](#footnote-ref-2)
