# Supplementary material for: Brucellosis as an Emerging Threat in Developing Economies: Lessons from Nigeria
Source: PLoS Negl Trop Dis. 2014 Jul 24;8(7):e3008. doi: 10.1371/journal.pntd.0003008 (PMC4109902; doi:10.1371/journal.pntd.0003008)
Supplement: Table S9 — Brucellosis bacteriology studies in cattle. (DOCX) [file pntd.0003008.s009.docx]

| **Reference** | **Origin of samples** | **Region** | **State**  **(City)^^[[1]](#footnote-1)^^** | **Period of**  **sampling^[[2]](#footnote-2)^** | **Media** | **Biotyping** | **Type of samples** | **n** | **Culture positive** | **Isolate** | **Biotype/**  **biovar** | **n** | **Comments** |  |
| --- | --- | --- | --- | --- | --- | --- | --- | --- | --- | --- | --- | --- | --- | --- |
| Ocholi et al., 2005 | Privately-owned farm |  | Bauchi  (Toro) | 2003 | FSM | CO_2_ dependence  H_2_S production  Urease  Monospecific sera  Growth on dyes  Phage sensitivity | Milk | 5 | 2 | *B. abortus* | 1 | 2 | Same isolate as (Ocholi et al. 2004) |  |
| Ocholi et al., 2004 | Private farm with horses, cattle, sheep and goats | North | NS | 2004 | NS | NS | Milk | 12 | 2 | *B. abortus* | 1 | 2 | Same isolate as (Ocholi et al. 2004) |  |
| Ocholi, et al. 2004 | NS | North | Taraba  Plateau  Adamawa  Bauchi  Nassawara  Sokoto  Kaduna  Kano  Sokoto  Kogi  Enugu | 2004 | FSM | CO_2_ dependence  H_2_S production  Urease  Monospecific sera  Growth on dyes  Phage sensitivity | Foetus  Vaginal swabs  Milk  Hygroma fluids  Blood | 10  448  139  9  94 | 2  5  12  6  0 | *B. abortus* | 1 | 17 | Isolates from:  Taraba  Plateau  Adamawa  Bauchi  Nassawara  Sokoto |  |
| Bale & Kumi-Diaka, 1981 | LIBCs | North | Kano | 1981 | BSL liquid medium then FSM | CO_2_ dependence  H_2_S production  Urease, Oxidase, Catalase  Monospecific sera  Growth on dyes  Phage sensitivity | Semen  Testicular exudate  Abomasal contents  Foetus heart blood  Milk aborted cattle  Hygroma fluid | 59  1  2  1  1  1 | 7  1  2  1  0  0 | *B. abortus*  *B. abortus*  *B. abortus*  *B. abortus* | 1  3  4  NS | 5  2  1  3 |  |  |
| Pullan, 1980 | Settled Fulani & indigene herds | North | Jos Plateau | 1975-1976 | NS | ND | Milk | NS | 3 | NA | NA | NA |  |  |
| Eze, 1978 | Government & private farms | North | Plateau  Niger  Borno  Kano | 1974-1976 | SDAA | CO_2_ dependence  H_2_S production  Monospecific sera  Growth on dyes  Phage sensitivity | Foetus  Milk  Hygroma fluid  Vaginal swabs | 19  911  10  83 | 0  16  4  0 | *B. abortus*  *B. abortus* | 1  2 | 19  1 | Isolates found in each state |  |
| Esuruoso, 1974b | Range cattle | West | Western  (Fashola) | 1968-1972 | NS | ND | Foetus | NS | 0 | NA | NA | NA | Isolation from and seroconversion of inoculated guinea pigs |  |
| Esuruoso & Van Blake, 1972 | University herd | West | Ibadan | 1972 | SDA | ND | Foetus | 6 | 0 | NA | NA | NA | Rabbits gave high SAT titres in month post-innoculation |  |

LIBCs-livestock investigation and breeding centres, NS- not specified, BSL- Brodie & Sinton’s liquid medium, FSM- Farrell solid medium, SDAA- serum dextrose antibiotic agar, SDA- serum dextrose agar, NA- not applicable, ND- not done

1. If the samples originate from more than one area, individual prevalence for each area is reported, if not, the overall state/region prevalence is reported [↑](#footnote-ref-1)
2. When period of study not specified, year of publication used [↑](#footnote-ref-2)
