# Supplementary material for: Brucellosis as an Emerging Threat in Developing Economies: Lessons from Nigeria
Source: PLoS Negl Trop Dis. 2014 Jul 24;8(7):e3008. doi: 10.1371/journal.pntd.0003008 (PMC4109902; doi:10.1371/journal.pntd.0003008)
Supplement: Table S10 — Brucellosis serology studies in sheep and goats reared under intensive livestock systems. (DOCX) [file pntd.0003008.s010.docx]

| **Reference** | **Population** | **Sampling method** | **Sampling approach** | **Bias**  **(gaps in method description)** | **Diagnostic test^^[[1]](#footnote-1)^^**  **(cut-off)** | **Region** | **Location**  **State (city)** | **Period of**  **sampling^[[2]](#footnote-2)^** | **Sample size**  **(no.flocks)** | | **Prevalence**  **(flock prev.) %** | | **Comments** |
| --- | --- | --- | --- | --- | --- | --- | --- | --- | --- | --- | --- | --- | --- |
|  |  |  |  |  |  |  |  |  | **S** | **G** | **S** | **G** |  |
| Onoja et al., 2008 | Semi-intensively managed flock | NPS | Outbreak investigation | Flock investigated due to suspicion of brucellosis | RBT | North | (Zaria) | 2008 | 17 (1) |  | 76 (100) |  |  |
| Junaidu et al., 2008 | Prison farm | NPS | Outbreak investigation | Farm investigated due to abortion storm in cattle | RBT | North | Sokoto State | 2005 | 76 (1) | 91 (1) | 22.36 (100) | 20.76 (100) |  |
| Ocholi et al., 2005 | Commercial flock | NPS | Abortion investigation | Flock investigated due to abortion storm | RBT | North | Bauchi State (Toro) | 2005 | 28 (1) |  | 14 (100) |  | *B. abortus* biovar 1 isolated from 5/28 ewes in flock |
| Brisibe et al., 1993 | University farm | NPS | Investigation of poor reproductive performance | Flock investigated due to fertility problems | RBT | North | Borno State  (Maiduguri) | 1993 | 58 (1) | 33 (1) | 15.5 (100) | 33.3 (100) |  |
| Adams & McKay, 1966 | Government herds | NPS? | NS | Sampling prompted by abortion storm in cattle | RPT (1:5) | East | NS | 1962 | 43 (NS) | 110 (NS) | 0 (NS) | 0 (NS) |  |
| Okoh, 1980 | Government LIBC | NPS | Abortion investigation | Farm investigated due to abortion storm | SAT  (40iu) | North | Kano State  (Rano) | 1977 | 372 (1) |  | 14.5 (100) |  | Abortion storm thought to have been triggered by introduction of sheep that had been in contact with cattle (Friesans and Devons) infected with *B. abortus* |

NS- not specified, NPS- non-probability sampling, SAT- serum agglutination test, RBT- rose Bengal test, MRT- milk ring test, no. – number, Gov.- government, Prev.- prevalence, S- sheep, G- goat

1. One test seroprevalence value per study reported in this preferential test order: RBT, CT, CFT, RPT, SAT, MRT. For studies that do not report parallel test results, seroprevalence value obtained with tests used in series reported (see text). [↑](#footnote-ref-1)
2. When period of study not specified, year of publication used [↑](#footnote-ref-2)
