# Supplementary material for: Brucellosis as an Emerging Threat in Developing Economies: Lessons from Nigeria
Source: PLoS Negl Trop Dis. 2014 Jul 24;8(7):e3008. doi: 10.1371/journal.pntd.0003008 (PMC4109902; doi:10.1371/journal.pntd.0003008)
Supplement: Table S11 — Brucellosis serology studies in sheep and goats reared under extensive livestock systems. (DOCX) [file pntd.0003008.s011.docx]

| **Reference** | **Population** | **Sampling**  **method** | **Sampling**  **approach** | **Bias**  **(gaps in method description)** | **Diagnostic test^[[1]](#footnote-1)^**  **(cut-off)** | **Period of**  **sampling^[[2]](#footnote-2)^** | **Region** | **Location**  **State/city/*LGA*^[[3]](#footnote-3)^** | **Sample size**  **(no.flocks)** | | **Prevalence**  **(flock prev.)**  **%** | | **Comments** |
| --- | --- | --- | --- | --- | --- | --- | --- | --- | --- | --- | --- | --- | --- |
|  |  |  |  |  |  |  |  |  | **S** | **G** | **S** | **G** |  |
| Kaltungo et al., 2013 | Pastoral and village-level goats | PS?^[[4]](#footnote-4)^ | Random selection of 4/7 LGAs  Random selection of flocks | Method for random selection of flocks not described | RBT | 2012 | North | Kaduna North State  *Ikara*  *Makarfi*  *Sabon Gari*  *Soba* |  | 442 (NS)  *83 (NS)*  *193 (NS)*  *56 (NS)*  *110 (NS)* |  | 25.8 (NS)  *26.5 (NS)*  *28.5 (NS)*  *28.6 (NS)*  *19.1 (NS)* | Overall prev. of 2.5% based on confirmation with LFA |
| Brisibe et al., 1996 | Nomadic and sedentary flocks | NPS | NS, sampling of all small ruminants in small flocks & 50% in large flocks | (Method not well characterised) | RBT | 1996 | North | Borno State, Maiduguri  Yobe State, Damaturu | 210 (NS) | 201 (NS) | 4.8 (NS) | 6.0 (NS) |  |

NS- not specified, NPS- non-probability sampling, PS- probability sampling, RBT- rose Bengal test, LFA- lateral flow assay, no. - number, prev.- prevalence, S- sheep, G- goat

1. If more than one diagnostic test has been used has been used, prevalence values for one test only selected to enable comparison of prevalence between studies [↑](#footnote-ref-1)
2. When period of study not specified, year of publication used [↑](#footnote-ref-2)
3. If the samples originate from more than one area, individual prevalence for each area is reported if the information is available [↑](#footnote-ref-3)
4. PS? Denotes that the sampling method is not well described but that probability sampling in most likelihood applies [↑](#footnote-ref-4)
