# Supplementary material for: Brucellosis as an Emerging Threat in Developing Economies: Lessons from Nigeria
Source: PLoS Negl Trop Dis. 2014 Jul 24;8(7):e3008. doi: 10.1371/journal.pntd.0003008 (PMC4109902; doi:10.1371/journal.pntd.0003008)
Supplement: Table S12 — Brucellosis serology studies in sheep and goats undertaken in extensive and intensive livestock systems in parallel. (DOCX) [file pntd.0003008.s012.docx]

| **Reference** | **Population** | **Sampling method** | **Sampling approach** | **Bias**  **(gaps in method description)** | **Diagnostic test^[[1]](#footnote-1)^**  **(cut-off)** | **Period of**  **sampling^[[2]](#footnote-2)^** | **Region** | **Location^3^** | **Livestock system** | **Sample size**  **(no. flocks)** | | **Prevalence**  **(flock prev.) %** | | **Comments** |
| --- | --- | --- | --- | --- | --- | --- | --- | --- | --- | --- | --- | --- | --- | --- |
|  |  |  |  |  |  |  |  |  |  | **S** | **G** | **S** | **G** |  |
| Bertu et al., 2010 | Fulani and local flocks, private and government semi-intensive flocks | NPS? | Clutch (cluster?) sampling? | (Sampling method not well characterised) | RBT | 2010 | North | Plateau State  *Bassa*  *Bokkos*  *Jos-North*  *Jos-South*  *Langtang North*  *Mangu*  *Panskshin*  *Q/Pan*  *Shendam* | COM  INT  EXT  INT  INT  EXT  INT  EXT  EXT  EXT | 496 (NS)  16 (NS)  91 (NS)  98 (NS)  91 (NS)  50 (NS)  94 (NS)  9 (NS)  17 (NS)  30 (NS) | 851 (NS)  71 (NS)  81 (NS)  157 (NS)  180 (NS)  50 (NS)  147 (NS)  64 (NS)  43 (NS)  58 (NS) | 9.3 (NS)  0 (NS)  11.0 (NS)  7.1 (NS)  13.2 (NS)  10 (NS)  6.4 (NS)  11.1 (NS)  5.9 (NS)  13.3 (NS) | 10.1 (NS)  5.6 (NS)  7.4 (NS)  12.1 (NS)  5.0 (NS)  8.0 (NS)  15.6 (NS)  14.1 (NS)  16.3 (NS)  8.6 (NS) | Plateau North= intro of animals from outside state, intensive farms  Plateau Centre & South= free-range management |
| Bale et al., 1982 | Institutional flocks, government & quasi-gov, farms, Fulani flocks, local flocks | NPS | NS | (Sampling method not characterised) | RBT | 1982 | North | NS | EXT  INT | 324 (NS)  382 (NS) | 261 (NS)  498 (NS) | 5.2 (NS)  21.4 (NS) | 5.7 (NS)  20.6 (NS) |  |
| Kramer et al., 1967 | Government ‘improved’ farms  Small unimproved farms | NPS?^4^ | Health assessment of 6/8 improved farms in area | Purposive sampling of government establishments & unimproved farms | SAT  (1:100) | 1967 | East | NS | EXT  INT | 49 (NS)  53 (NS) |  | 2.0 (NS)  0 (NS) |  |  |

NS- not specified, NPS- non-probability sampling, SAT- serum agglutination test, RBT- rose Bengal test, no. – number, EXT- extensive, INT- intensive, COM- combined, prev.- prevalence, S- sheep, G- goat

1. One test seroprevalence value per study reported in this preferential test order: RBT, CT, CFT, RPT, SAT, MRT. For studies that do not report parallel test results, seroprevalence value obtained with tests used in series reported (see text). [↑](#footnote-ref-1)
2. When period of study not specified, year of publication used.

   3 If the samples originate from more than one area, individual prevalence for each area is reported if the information is available.

   ^4^ NPS? Denotes that the method is not described but that non-probability sampling in most likelihood applies. [↑](#footnote-ref-2)
