# Supplementary material for: Brucellosis as an Emerging Threat in Developing Economies: Lessons from Nigeria
Source: PLoS Negl Trop Dis. 2014 Jul 24;8(7):e3008. doi: 10.1371/journal.pntd.0003008 (PMC4109902; doi:10.1371/journal.pntd.0003008)
Supplement: Table S13 — Brucellosis serology studies in sheep and goats under non-specified livestock systems. (DOCX) [file pntd.0003008.s013.docx]

| **Reference** | **Population** | **Sampling method** | **Sampling approach** | **Bias**  **(gap in method description)** | **Diagnostic test^^[[1]](#footnote-1)^^**  **(cut-off)** | **Period of**  **sampling^[[2]](#footnote-2)^** | **Region** | **Location**  **State (city)^^[[3]](#footnote-3)^^** | **Sample size**  **(no.flocks)** | | **Prevalence**  **(flock prev.) %** | | **Comments** |
| --- | --- | --- | --- | --- | --- | --- | --- | --- | --- | --- | --- | --- | --- |
|  |  |  |  |  |  |  |  |  | **S** | **G** | **S** | **G** |  |
| Brisibe et al., 1993 | Government vet. clinic | NPS | Convenience sampling | (Method not characterised) | RBT | 1993 | North | Borno State  (Maiduguri) | 50 (NS) | 28 (NS) | 2.0 (NS) | 0 (0) |  |
| Falade et al., 1974 | Goats from vet clinics in south, private households in west | NPS?^[[4]](#footnote-4)^ | NS | (Method not characterised) | SAT  (50iu) | 1974 | West  South | Western State  Mid Western State  Rivers State  South Eastern State |  | 759 (NS)  7 (NS)  19 (NS)  9 (NS) |  | 5.42 (NS)  0  0  0 |  |
| Kramer et al., 1967 | Government ‘improved’ farms  Small unimproved farms | NPS? | Health assessment of 6/8 improved farms in area | Purposive sampling of government establishments & unimproved farms | SAT  (1:100) | 1967 | East | NS |  | 163 (NS) |  | 0.6 (NS) | Positive goat from Northern Province  Co-rearing with cattle |

NS- not specified, NPS- non-probability sampling, RBT- rose Bengal test, SAT- serum agglutination test, no. – number, prev- prevalence

1. One test seroprevalence value per study reported in this preferential test order: RBT, CT, CFT, RPT, SAT, MRT. For studies that do not report parallel test results, seroprevalence value obtained with tests used in series reported (see text). [↑](#footnote-ref-1)
2. When period of study not specified, year of publication used [↑](#footnote-ref-2)
3. If the samples originate from more than one area, individual prevalence for each area is reported, if not, the overall state prevalence is reported [↑](#footnote-ref-3)
4. NPS? Denotes that the method is not described but that non-probability sampling in most likelihood applies [↑](#footnote-ref-4)
