# Supplementary material for: Brucellosis as an Emerging Threat in Developing Economies: Lessons from Nigeria
Source: PLoS Negl Trop Dis. 2014 Jul 24;8(7):e3008. doi: 10.1371/journal.pntd.0003008 (PMC4109902; doi:10.1371/journal.pntd.0003008)
Supplement: Table S14 — Brucellosis abattoir serology studies in sheep and goats. (DOCX) [file pntd.0003008.s014.docx]

| **Reference** | **Origin of animals** | **Diagnostic test^^[[1]](#footnote-1)^^**  **(cut-off)** | **Period of**  **sampling^[[2]](#footnote-2)^** | **Region** | **City (State)** | **Name of abattoir**  **/market** | **n** | | **Prev. (%)** | | **Comments** |
| --- | --- | --- | --- | --- | --- | --- | --- | --- | --- | --- | --- |
|  |  |  |  |  |  |  | **S** | **G** | **S** | **G** |  |
| Gusi et al., 2010 | NS (Plateau State?) | RBT | 2009 | North | Jos | Jos Main AB | 128 | 187 | 5.5 | 7.5 |  |
| Junaidu et al., 2010 | NS (Sokoto State?, North?) | RBT | 2008-2009 | North | Sokoto | Sokoto Metrop. AB |  | 532 |  | 12.0 |  |
| Tijjani et al., 2009 | Yobe State (North?) | RBT | 2007 | East | Damaturu | Damaturu AB | 300 | 400 | 6.0 | 14.0 |  |
| Cadmus et al, 2006 | North & Chad, Niger, Mali, Burk. Fas., Cameroon | RBT | 2004 | West | Ibadan | Bodija Municipal AB | 54 | 496 | 0 | 0.86 |  |
| Brisibe et al., 1993 | Borno State? (North?) | RBT | 1993 | North | Maiduguri | Maiduguri AB | 102 | 140 | 0 | 0.7 |  |
| Okewole et al., 1988 | Kano, Borno, Bauchi & Plateau States  (from local farms, free-range grazing) | RBT | 1982-1983 | North | Jos | Jos AB | 83 | 415 | 2.4 | 0.7 |  |
| Bale et al., 1982 | ‘Far and near’ including neighbouring countries,  considered as transient animals | RBT | 1982 | North | NS | NS AB | 179 | 1015 | 14.5 | 16.6 |  |
| Falade, 1980 | NS (North?) | RBT | 1980 | North  West | Kano, Bodija, Oja-Oba | NS AB & MK |  | 705 |  | 0.3 |  |
| Falade et al., 1974 | Nomadic flocks North | SAT  (50iu) | 1978 | North | (Kano)  (North Western)  (North Central)  (Kwara)  (North Eastern)  (Benue Plateau ) | NS AB/MK  NS AB/MK  NS AB/MK  NS AB/MK  NS AB/MK  NS AB/MK |  | 1251  13  48  107  248  89 |  | 4.6  0  4.2  3.8  0.8  3.4 |  |
| Shehu et al., 1999 | NS (Bauchi State?; neighbouring countries) | SAT (NS) | 1999 | North | Bauchi | Bauchi Metrop. AB | 530 | 1010 | 6.6 | 4.7 | 2 game reserves in Bauchi- wildlife reservoir? |

NS- not specified, SAT- serum agglutination test, RBT- rose Bengal test, AB- abattoir, MK- market, Burk. Fas.- Burkina Faso, Prev.- prevalence, S- sheep, G- goat

1. One test seroprevalence value per study reported in this preferential test order: RBT, CT, CFT, RPT, SAT, MRT. For studies that do not report parallel test results, seroprevalence value obtained with tests used in series reported (see text). [↑](#footnote-ref-1)
2. When period of study not specified, year of publication used. [↑](#footnote-ref-2)
