# Supplementary material for: Brucellosis as an Emerging Threat in Developing Economies: Lessons from Nigeria
Source: PLoS Negl Trop Dis. 2014 Jul 24;8(7):e3008. doi: 10.1371/journal.pntd.0003008 (PMC4109902; doi:10.1371/journal.pntd.0003008)
Supplement: Table S15 — Brucellosis bacteriology studies in sheep and goats. (DOCX) [file pntd.0003008.s015.docx]

| **Reference** | **Origin of samples** | **Region** | **State**  **(City)** | **Period of**  **sampling^[[1]](#footnote-1)^** | **Media** | **Biotyping** | **Type of samples** | **No. samples** | | **Culture positive** | | **Isolate** | **Biotype/biovar** | **No. isolates** | **Comments** |
| --- | --- | --- | --- | --- | --- | --- | --- | --- | --- | --- | --- | --- | --- | --- | --- |
|  |  |  |  |  |  |  |  | **S** | **G** | **S** | **G** |  |  |  |  |
| Ate et al., 2011 | NS | North | (Zaria) | 2011 | NS | NS | Foetus | 1 |  | 1 |  | *B.ovis?* | NA | 1 | In absence of description of biotyping method isolation of *B. ovis* questionable |
| Onojo, 2008 | Privately-owned farm | North | (Zaria) | 2008 | SDA, BA | NA | Hygroma fluid | 3 |  | 0 |  | NA | NA | NA | Serological evidence of brucellosis in flock |
| Ocholi et al., 2005 | Privately-owned farm | North | Bauchi  (Toro) | 2003 | FSM | CO_2_ dependence  H_2_S production  Urease  Monospecific sera  Growth on dyes  Phage sensitivity | Milk  Vaginal swabs | 5  7 |  | 2  0 |  | *B. abortus* | 1 | 5 (S) | Same isolates as (Ocholi et al. 2004) |
| Ocholi, et al. 2004 | NS | North | Plateau  Bauchi  Adamawa  Bornu | 2004 | FSM | CO_2_ dependence  H_2_S production  Urease  Monospecific sera  Growth on dyes  Phage sensitivity | Foetus  Vaginal swabs  Milk | 2  50  13 | 0  7  8 | NA  1  2 | NA  0  0 | *B. abortus* | 1 | 3 (S) | Isolates from:  Bauchi  Milk samples/vaginal swabs |
| Bale et al., 2003 | 7 Government farms | North | (Zaria)  (Shika)  (Kukar Aljana)  (Katsina)  (Rimi)  (Tuma)  (Rano)  (Dangora) |  | BSL  FSM  SDA | CO_2_ dependence  H_2_S production  Urease, Oxidase, Catalase  Monospecific sera  Growth on dyes  Phage sensitivity  Indole, Methyl-red  Voges Proskauer, Nitrate  Mod.ZN  Agglut. with acriflavine | Milk | 277 | 141 | 4 | 6 | *B. melitensis?* | ?* | 10 | * Could not biotype by phage typing as were rough strains  *Brucella* isolated from 4/7 farms\| |
| Falade, 1981 | Abattoirs, markets, private households | West | Oyo State | 1981 | SDAA | CO_2_ dependence  H_2_S production  Urease  Monospecific sera  Growth on dyes  Phage sensitivity | Foetuses  Vaginal swabs  Milk* |  | 146  5  269 |  | 3  1  26 | *B. melitensis*  *B. abortus* | 1  1 | 22  8 | *MRT positive |
| Okoh, 1980 | Government LIBC | North | Kano State (Rano) | 1977 | NS | NS | Milk | 22 |  | 5 |  | *B. abortus?* | ND | NS |  |
| Eze, 1978 | Government & private farms | North | Plateau  Niger  Borno  Kano | 1974-1976 | SDAA | CO_2_ dependence  H_2_S production  Monospecific sera  Growth on dyes  Phage sensitivity | Milk  Vaginal swabs | 31  0 | 71  15 | 0  NA | 0  0 | NA | NA | NA | Isolates obtained from cattle |

LIBC-livestock investigation and breeding centre, NS- not specified, BSL- Brodie & Sinton’s liquid medium, FSM- Farrell solid medium, SDAA- serum dextrose antibiotic agar, SDA- serum dextrose agar, BA- *Brucella* agar, NA- not applicable, ND- not done, S- sheep, G- goat

1. When period of study not specified, year of publication used [↑](#footnote-ref-1)
